# SeqCode: a nomenclatural code for prokaryotes described from sequence data

**DOI:** 10.1038/s41564-022-01214-9

**Published:** 2022-09-19

**Authors:** Brian P. Hedlund, Maria Chuvochina, Philip Hugenholtz, Konstantinos T. Konstantinidis, Alison E. Murray, Marike Palmer, Donovan H. Parks, Alexander J. Probst, Anna-Louise Reysenbach, Luis M. Rodriguez-R, Ramon Rossello-Mora, Iain C. Sutcliffe, Stephanus N. Venter, William B. Whitman

**Affiliations:** 1 School of Life Sciences, University of Nevada, Las Vegas, Las Vegas, NV USA; 2 The University of Queensland, School of Chemistry and Molecular Biosciences, Australian Centre for Ecogenomics, Brisbane, Queensland Australia; 3 School of Civil and Environmental Engineering, Georgia Tech, Atlanta, GA USA; 4 Division of Earth and Ecosystem Sciences, Desert Research Institute, Reno, NV USA; 5 Department of Chemistry, Environmental Microbiology and Biotechnology (EMB), Group for Aquatic Microbial Ecology and Centre of Water and Environmental Research (ZWU), University of Duisburg-Essen, Essen, Germany; 6 Biology Department, Portland State University, Portland, OR USA; 7 Department of Microbiology and Digital Science Center (DiSC), University of Innsbruck, Innsbruck, Austria; 8 Marine Microbiology Group, Department of Animal and Microbial Diversity, Mediterranean Institute of Advanced Studies (CSIC-UIB), Esporles, Spain; 9 Faculty of Health & Life Sciences, Northumbria University, Newcastle upon Tyne, UK; 10 Department of Biochemistry, Genetics and Microbiology, University of Pretoria, Pretoria, South Africa; 11 Department of Microbiology, University of Georgia, Athens, GA USA

**Keywords:** Bacteriology, Environmental microbiology, Classification and taxonomy

## Abstract

Most prokaryotes are not available as pure cultures and therefore ineligible for naming under the rules and recommendations of the International Code of Nomenclature of Prokaryotes (ICNP). Here we summarize the development of the SeqCode, a code of nomenclature under which genome sequences serve as nomenclatural types. This code enables valid publication of names of prokaryotes based upon isolate genome, metagenome-assembled genome or single-amplified genome sequences. Otherwise, it is similar to the ICNP with regard to the formation of names and rules of priority. It operates through the SeqCode Registry (https://seqco.de/), a registration portal through which names and nomenclatural types are registered, validated and linked to metadata. We describe the two paths currently available within SeqCode to register and validate names, including *Candidatus* names, and provide examples for both. Recommendations on minimal standards for DNA sequences are provided. Thus, the SeqCode provides a reproducible and objective framework for the nomenclature of all prokaryotes regardless of cultivability and facilitates communication across microbiological disciplines.

## Abstract (web summary)

SeqCode is the result of a community effort to unify nomenclature for uncultured and cultured prokaryotes using genome sequences.

## Main

It is widely recognized that the requirement of the International Code of Nomenclature of Prokaryotes (ICNP) for deposition of axenic and viable cultures as nomenclatural types has hindered the development of a nomenclature for uncultured and fastidious cultured prokaryotes (archaea and bacteria) and thus effective communication of microbial diversity^1–3^. As-yet-uncultivated taxa account for ~85% of the phylogenetic diversity of prokaryotes^4^ and named prokaryotes account for <0.2% of total species^5^. By excluding the uncultured majority, a substantial portion of the tree of life is relegated to poorly ordered, ambiguous and often synonymous names or alphanumeric codes. Most of these alphanumeric codes are of limited mnemonic value because each letter or number contributes to a limited memory or digit span^6^, whereas a taxonomic name can be remembered as a single word, especially if it is meaningful or familiar.

To address this problem, Konstantinidis et al.^1^ and subsequently Murray et al.^2^ proposed two paths, which were endorsed by 121 authors and signatories from 22 countries and six continents^2^. Initial ‘plan A’ was based on proposals that DNA sequences could serve as nomenclatural types and be incorporated into the existing ICNP infrastructure^7^. However, the International Committee on Systematics of Prokaryotes (ICSP) rejected that proposal^8^, thus triggering ‘plan B’, which called for a new code of nomenclature^2^.

## Results

Recognizing the importance of further community engagement in the implementation of ‘plan B’, an ad hoc SeqCode Organizing Committee held a series of online workshops (https://www.isme-microbes.org/reports-sponsored-events) that garnered over 848 registrants from a broad range of microbiology disciplines, from 42 countries and 6 continents, as described in the Methods. Over 90% of participants reported that they would use a new code that accepts DNA sequences as types (https://www.isme-microbes.org/sites/default/files/reports/Path_forward_Naming_Uncultivated.pdf). Given strong participation and near-unanimous support, the SeqCode Organizing Committee deliberated carefully and acted on a variety of community recommendations as described in the Methods. The result was the writing of the SeqCode (formally The Code of Nomenclature of Prokaryotes Described from Sequence Data; Supplementary Information) and progress on systems to implement it. These actions initiated a process with the goal of SeqCode implementation through community support and action (Table 1), with this publication serving as a crucial but early step.

**Table 1:** Plan of action for the successful implementation of the SeqCode with community engagement

Step	Notes
Initial draft of SeqCode	Presented in online discussions in early 2021 and revised by the SeqCode Organizing Committee.
Preparation of SeqCode v.1.0	Proposed herein; additional changes made reflecting discussions of the preprint in online discussion forums and reviewer comments.
Construction of SeqCode Registry	Currently being constructed. Needs testing and user feedback. Contingent upon community support, will incorporate automatic tools to evaluate genome quality.
Formation of administrative body of the SeqCode	Online discussion forum currently available for discussion of a proposed administrative structure including the SeqCode Committee, Executive Board and Reconciliation Committee. Needed to ensure longevity, future amendments of the code and funding strategies.
Add *Candidatus* taxa to Registry	Validly publish backlog of *Candidatus* names already described in the literature by entry into the SeqCode Registry.
Development of path 3 to validate names	Work with journals to develop an integrated review system for manuscripts and SeqCode Registry.
Write SeqCode v.2.0	SeqCode is a living document. Experience will lead to ideas for improvements.
Merge the nomenclature of the SeqCode with that of the ICNP	Will maximize the synergies between the laboratory and field disciplines in microbiology.

Links to publications, preprints, discussion forums and other information can be found at www.isme-microbes.org/seqcode-initiative

The SeqCode uses genome sequence data as common currency for typification of both cultivated and uncultivated microorganisms and follows rules similar to those of the ICNP for priority^9^. In essence, the rules of both codes state that the earliest validly published name for a taxon in a particular position is the correct name (has priority), observing historical precedent and stabilizing nomenclature. The SeqCode also recognizes the priority of names validly published under the rules of the ICNP provided they do not violate the priority of names published under the SeqCode, thus minimizing divergence between the systems.

### Name validation through the SeqCode Registry

Taxonomic names validated under the SeqCode will be captured in the SeqCode Registry, a registration web portal through which names and nomenclatural types are registered, validated and linked to metadata. The SeqCode Registry supports three main objectives: (1) the registration and evaluation of names to be proposed in accordance with the SeqCode; (2) the automated identification of *Candidatus* names currently used in the literature so that many of them may be normalized and standardized through validation under the SeqCode; and (3) the maintenance of a standardized, publicly available list of names validated under the SeqCode, along with key links and machine-readable metadata. While still under development, a draft version is currently available at https://seqco.de/. All of its public data are accessible and reusable through the Creative Commons Attribution 4.0 License, except where otherwise noted, and the underlying code is released as open source under the terms of the Massachusetts Institute of Technology License. When completed, the SeqCode Registry will provide user-friendly, graphical interface access to its resources as well as computer-readable entries in JavaScript Object Notation format for easy integration by third-party services. Examples of the system’s use are provided below and in the Supplementary Information for the registration of names under different publication circumstances.

**Fig. 1:**
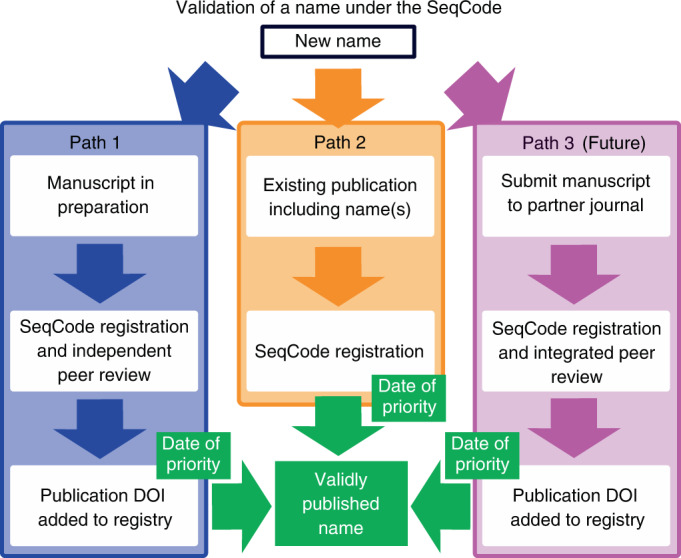
Validation process of a name under the SeqCode. Currently, two mechanisms exist, with a third possible in the future. In the recommended mechanism (blue arrows, path 1), draft registration of the name and entry of metadata into the SeqCode Registry occurs concurrently with preparation of the effective publication. Within the Registry, data quality and name synonymy checks in conjunction with curator review take place as described in Tables 2 and 3, leading to provisional acceptance of proposals that comply with SeqCode rules. This procedure ensures data quality and avoids requiring errata after publication for corrections. Entry of the DOI of the publication into the Registry marks the time and date of priority. Because the SeqCode requires that the earliest name of a taxon be used, the date of priority establishes the precedence of this name as the only valid name for the taxon. The second (orange arrows, path 2) is for names that are already published, such as *Candidatus* names. The name and metadata are entered into the Registry. Automated checks and SeqCode curators review compliance and acceptance of the proposal completes registration and marks the time and date of priority. At that point, the *Candidatus* designation can be removed. The third mechanism could be developed in partnership with one or more journals in the future (pink arrows, path 3). It would involve simultaneous peer review and Registry curator review as an integrated path to the validation of proposed names. Issuance of the DOI of the accepted paper marks the time and date of priority. Please see the text and Supplementary Information for concrete examples of registration through paths 1 and 2.

Currently, two different mechanisms to register and validate names are available through the SeqCode Registry (Fig. 1); a third mechanism may be possible in the future. In the best-case scenario, data will be entered and reviewed before publication through a preregistration process that takes place before initial submission or resubmission of a manuscript (Fig. 1, left or blue arrows, path 1). This route allows the SeqCode Registry to perform automated checks and provide curator input, both of which serve as resources to guide the user community. By providing these prechecks, path 1 serves two important roles as follows. (1) Automated checks and curator input during preregistration can prevent mistakes such as synonymy or problems with Latinization before names are published and thus prevent confusion resulting from name changes after publication. This process is thus somewhat similar to manual nomenclatural checks during peer review at the *International Journal of Systematic and Evolutionary Microbiology* (*IJSEM*). However, by automating the process as much as possible, the aim is to maximize speed and scalability and minimize human error. Similarly, data quality checks guide the user community by ensuring that genomic data serving as nomenclatural types are of sufficient quality. Currently genome quality and completeness data are entered by the user and checked against requirements and recommendations, although in the future these checks will be automated. (2) SeqCode identifier Uniform Resource Locators (URLs) generated during preregistration can be included in manuscripts that are submitted as the effective publication (the publication in which new names are proposed). These URLs allow peer reviewers and editors to access the preregistered names to ensure they have passed SeqCode checks. This process should improve and simplify peer review of new names and associated genomes because approval by the SeqCode Registry at the preregistration phase can provide confidence that the names are free of problems such as synonymy and poor Latinization and that the sequence data serving as the nomenclatural type are of sufficient quality. It should also be noted that minor orthographic variants of names that are validated under the SeqCode can be proposed by anyone at any time within the SeqCode Registry without publishing errata, which is also aimed at minimizing workload and confusion. Decisions on these orthographic variants will be refereed by curators. Under path 1, the completion of the registration process and thus date of priority of a name, is the date on which the Digital Object Identifier (DOI) is entered in the SeqCode Registry. This would normally be done by authors but, if SeqCode identifier URLs are used in the effective publication, then the DOI will be automatically captured by the SeqCode Registry once the manuscript is published, completing the registration process.

The second mechanism (middle or orange arrows, path 2) allows registration and validation of names that are already published, including *Candidatus* names. The name and metadata are entered into the Registry and screened by the same automated checks implemented under path 1. Then, SeqCode curators review the names and acceptance of the entry completes registration and marks the date of priority of the name. At that point, the name is valid and the *Candidatus* designation can be removed. We note that path 2 is less desirable than path 1 because problems with nomenclature or genome quality would not be flagged and corrected before publication of the names. As such, names published in the literature may ultimately be emended or invalid under the SeqCode; however, as described above, the SeqCode and Registry are deliberately designed to be as flexible as possible to best serve the community. This is possible because the SeqCode Registry is simultaneously the registration and validation system and the official and up-to-date listing of names validated under the SeqCode. The third mechanism (right or pink arrows, path 3) would involve simultaneous peer review and Registry curator review as an integrated path to the validation of proposed names, similar to the integrated review system of the *IJSEM*, which serves names proposed under the ICNP.

### Application of the SeqCode before publication through path 1

A concrete example of how this process might work is described below for *Wolframiiraptor gerlachensis* and related taxa and in the Supplementary Information. Briefly, several authors of the SeqCode (M.P., A.-L.R. and B.H.) recently completed a combined cultivation/metagenomics study of a previously undescribed group of Archaea in the Genome Taxonomy Database (GTDB) family designated as NZ13-MGT within the phylum Thermoproteota^10^, also previously discussed in the literature as ‘Aigarchaeota’ groups 4, 5 and 7 (refs. ^11,12^). The study initially focused on anaerobic enrichment cultures from sediments of Great Boiling Spring, Nevada, United States, containing a single member of the taxon, which was shown to require tungsten for growth on corn stover or a sugar mix under fermentative conditions. Fluorescence in situ hybridization combined with nanometre-scale secondary ion mass spectrometry was then used to confirm xylose as the preferred substrate. The taxon was represented by a single high-quality metagenome-assembled genome (MAG), although that MAG formed a >99.5% average nucleotide identity (ANI) cluster with MAGs of lower quality from separate samples of the same enrichment culture and sediments from which the enrichment culture was derived. To expand the study, 77 additional high-quality MAGs assigned to the GTDB family NZ13-MGT by GTDB-Tk^13^ were assembled from metagenomes from other terrestrial and marine hydrothermal systems.

It is recommended in Table 2 that species or subspecies named under the SeqCode include more than one genome. This parallels the general recommendation under the ICNP to characterize multiple strains for proposals of new taxonomic names and is especially important for MAGs and single-amplified genomes (SAGs) because of challenges associated with accurately binning metagenomic data and the low completeness of most SAGs. Here, FastANI^14^ was used to dereplicate the 78 high-quality MAGs into 11 >95% ANI clusters (species clusters^14,15^), and phylogenetic analyses of concatenated marker gene sets confirmed that each ANI cluster was monophyletic. In total, 9 of the species clusters were represented by 2–16 high-quality MAGs (after Bowers et al.^16^) from metagenomes from different sampling dates and/or geothermal springs. Comparison of the multiple MAGs per species cluster allowed assessment of: (1) monophyly of each species by using a multiple marker gene set; (2) the true presence of multiple copies of normally single-copy, conserved marker genes and true absence of conserved marker genes used to assess genome completeness and contamination; (3) the existence of homologues of genes encoding important functions (in this case, tungstate transporters, tungstoenzymes and genes related to energy conservation); (4) shared gene content in general; and (5) similar genome content and size for the genomes within a species. These comparisons strengthened conclusions about the proposed mode of energy conservation, evolution of the organisms and their enzyme systems and allowed identification and rejection of problematic MAGs. We note that the MAGs from several GTDB species representatives were detected and analysed phylogenetically but most were not of sufficient quality to name under the SeqCode (Table 3).

**Table 2:** Requirements and recommendations for publication of new species names under the SeqCode

To be included in the effective publication^a^
**Required**
Name
**Recommended**
Etymology
Name formed with mnemonic cues
Interpretation of biological properties inferred or demonstrated physiological traits and ecological information, such as habitat, in the manuscript body and/or protologue
Designated type genome assembly (for example, INSDC accession) and access to raw data (for example, Sequence Read Archive (SRA) accession)
Include as much metadata as possible in the INSDC submission^24^
Provide evidence of the species, taxonomic rank and position, including the uniqueness of the species with respect to existing named species and justify the taxonomic rank and position. Check for congruence between the genome and 16S rRNA taxonomic assignments^14,26,27^
For MAGs and SAGs, compare multiple high-quality genomes representing the species in more than one sample. Genomic assemblies from multiple samples can support the non-chimaeric nature of MAGs and provide confidence in the assembly for both MAGs and SAGs
**Rationale:** Initial requirements encourage wide participation from many microbiological disciplines and enable validation of names published before the SeqCode. Critical data will be captured in the SeqCode Registry. Some recommendations could become requirements in the future.

^a^Under the SeqCode, as under all major codes of nomenclature, the term effective publication refers to the publication in which new taxonomic names are proposed. Under the SeqCode, effective publications must be peer-reviewed.

Recommendations are suggested best practices to guide authors and peer reviewers to ensure high-quality data supporting species to be named. See text and Supplementary Information for examples.

In the end, type sequences meeting the data quality standards for the SeqCode (Table 3) were available for 11 species clusters, leading to proposals for 11 species names as well as their parent taxa under the SeqCode. Names were formed under the rules of Latin following general recommendations of Appendix 9 of the ICNP and other guidance^17^ and were checked by the nomenclature expert A. Oren. In the future, they would be checked by curators within the SeqCode Registry. The process for preregistration is described in detail in the Supplementary Information section entitled ‘SeqCode preregistration’. Following preregistration, the effective publication^10^ was submitted for peer review. The effective publication includes the following for each taxonomic name: (1) clear designation of the nomenclatural type; (2) designation of the taxonomic rank; and (3) etymology of the new name (Table 2). The nomenclature proposals were presented within protologues, examples of which for two taxonomic ranks are shown below. While protologues are not required under the SeqCode, they are useful for taxonomic descriptions because they compile the critical information in one place. Tables may also be used, examples of which are in the Supplementary Information. We note here that the SeqCode Registry produces protologues once preregistration is complete. Those protologues are useful for the scientific community as they can be linked via URLs within the effective publication, modified to serve as protologues in publications or accessed any time online within the Registry.

**Table 3:** Data quality and reporting requirements and recommendations for an isolate genome, MAG or SAG to serve as the nomenclatural type for a species named under the SeqCode

Data quality necessary for completion of SeqCode Registry^a^
**Required**
Type genome assembly quality for MAGs and SAGs: >90% complete and <5% contaminated (modified from Bowers et al. ^16^)
For isolates, read coverage ≥10× (Field et al. ^24^)
**Recommended**
16S rRNA genes >75% complete, passes chimaera checks
Agreement between genome and 16S rRNA taxonomic assignments
>80% of transfer RNAs present (modified from Bowers et al. ^16^)
High genome integrity (contig no. <100; N50 >25 kilobases (kb); largest contig >100 kb)
MAG/SAG read coverage ≥10×
**Data availability required for SeqCode Registry**
Type assembly available in INSDC databases
Raw data for type available in INSDC databases (for example, SRA)^b^
**Rationale:** Registry queries the INSDC databases to perform automatic checks of data quality

^a^Data quality will be assessed by automated pipelines or other approaches. Exceptions for lower data quality should be justified by authors in the effective publication.

^b^Not required for names effectively published before 1 January 2023 to allow for existing published names (for example, existing *Candidatus* names) and names currently undergoing peer review to be validated under the SeqCode.

Requirements will be checked as part of the validation process on the SeqCode Registry. Recommendations are suggested best practices to guide authors and peer reviewers to ensure high-quality data supporting species to be named. See text and Supplementary Information for examples.

In the effective publication^10^, names are proposed for the previously undescribed family *Wolframiiraptoraceae*, which is the parent taxon for the previously undescribed genus *Wolframiiraptor.* This family name replaces the GTDB designation NZ13-MGT and is described in the Supplementary Information. The protologue below describes the previously undescribed genus *Wolframiiraptor*. Note that for a genus, the nomenclatural type is a species, as in the ICNP. Notes explaining the elements of the protologue as they pertain to the principles, rules and recommendations of the SeqCode are shown in brackets.

*Wolframiiraptor* (Wolf.ra.mi.i.rap’tor N.L. neut. N. *wolframium*, tungsten; L. masc. n. *raptor*, snatcher or thief; N.L. masc. n. *Wolframiiraptor*, snatcher of tungsten). (This text designates the taxonomic rank (genus) and the etymology under SeqCode rules 26.4 and 26.5.)

Members of this genus have been identified from geothermal springs in the Great Basin and Yellowstone National Park, United States, and the Rehai Geothermal Field and the town of Dientan, Tengchong, China. Average amino acid identity (AAI) values among genomes representing separate species within the genus range between 81% and 90%. On the basis of ancestral state reconstruction analysis, likely losses of the genes encoding cytochrome *c* oxidase subunits, the aerobic carbon monoxide dehydrogenase large subunit and sulfide:quinone oxidoreductase (Sqr), indicate that members of this genus are probably strict anaerobes and are incapable of sulfide oxidation. Genomes of this genus encode a *tupA* subunit of the tungstate (Tup) ABC transporter and contain multiple genes encoding tungsten-dependent oxidoreductases, including three putative aldehyde:ferredoxin oxidoreductase (AOR)-like, one formaldehyde:ferredoxin oxidoreductase (FOR-like) and one glyceraldehyde-3-phosphate:ferredoxin oxidoreductase (GAPOR)-like proteins. This taxon is supported as a genus-level group by phylogenomics, AAI and relative evolutionary divergence. (This text includes a description of the taxon, following recommendation 26. Such text is recommended but not required under the SeqCode.)

The nomenclatural type of the genus is *Wolframiraptor gerlachensis*^Ts^. (This text designates the nomenclatural type under rule 26.3. Note that the nomenclatural type for rank of genus is a species, typically the first legitimate species in the genus. These dates are clearly shown in the SeqCode Registry. Rule 26.3 embodies principle 5 and serves to unambiguously identify the taxon. See rule 16 and rule 22. Note that genus names do not need to have a standard suffix like family, order and above but they should avoid suffixes used for other taxonomic ranks to prevent confusion. See rule 15. Under chapter 4, the superscript Ts can be added when this species is a nomenclatural type and the type of the species is a DNA sequence.)

The protologue below describes the previously undescribed species *W. gerlachensis*. Note that for a species, the nomenclatural type is a DNA sequence, typically a genome assembly (Table 3).

*W. gerlachensis*^Ts^ (ger.lach.en’sis N.L. masc. adj. *gerlachensis*, of Gerlach, the town where Great Boiling Spring is located in Nevada and where the samples containing this organism were obtained.) (This text designates the taxonomic rank (species) and the etymology under rules 26.4 and 26.5. Under chapter 4, the superscript Ts can be added to denote that this species is the type for the genus and its type is a DNA sequence.)

A MAG representing this species was recovered from metagenomic sequencing of a stable enrichment culture, established from an in situ corn stover enrichment from Great Boiling Spring, Nevada, United States. Enrichment and maintenance of this species within the mixed-culture community was optimal at an incubation temperature of 80 °C with lignocellulose or a mix of sugars as carbon sources under fermentative conditions, at circumneutral pH. This species was dependent on tungsten for growth; without tungsten added to the growth medium, the species was lost after several culture transfers. Additionally, transcripts for several tungstoenzymes conserved within the genus were present at high abundance during growth on corn stover, suggesting direct involvement of tungstoenzymes in fermentation of complex carbohydrates. Cells of this organism showed significant isotope enrichment when grown on isotopically labelled xylose-amended medium, with limited isotope enrichment during growth on medium amended with isotopically labelled amino acids, glucose, ribose or starch, indicating preferential assimilation of xylose. The type genome sequence of this species is 1,277,965 base pairs, consists of 27 contigs and has a G + C content of 52%. Completeness is estimated at 98.06% with 0.49% contamination, as estimated with CheckM. ANI comparisons between this genome and those of closely related species were below 86%, supporting the delineation of this taxon as unique and distinct from other species in the genus. (This text includes a description of the taxon, following recommendation 26. Such text is recommended but not required under the SeqCode.)

The genome Wger_A8^Ts^, available under the GenBank assembly accession number (GCA_021323375.2^Ts^), is the designated nomenclatural type for the species and was recovered from an enrichment culture, established from an in situ enrichment from Great Boiling Spring, Nevada, United States. (This text designates the nomenclatural type under rule 26.3. Note that the nomenclatural type for rank of species or subspecies is a DNA sequence, typically a genomic assembly. Rule 26.3 embodies principle 5 and serves to unambiguously identify the taxon. Metadata for this sequence is included in the GenBank entry. Under chapter 4, the superscript Ts can be added to denote that this genomic assembly is the nomenclatural type of the species.)

### Application of the SeqCode through path 2 for already published names, including *Candidatus* names

The SeqCode also enables registration of previously published names, such as *Candidatus* names that conform to its rules. *Candidatus* is a provisional status lacking priority and standing in nomenclature and is relegated to the non-legislative appendix 11 of the ICNP. It was developed for organisms for which ‘more than a mere nucleic acid sequence is available’^18^. Since its inception, visualization of the taxon in a natural sample has been recommended^18,19^ but this is rarely implemented. It has been argued that *Candidatus* names should be granted priority under the ICNP^20^; however, this proposal was also rejected by the ICSP^8^. As a result, many *Candidatus* names may prove to be ephemeral. Validation of these names under the SeqCode will give them priority and the *Candidatus* designation can be dropped (Fig. 1, path 2). These names are of special importance because a catalogue of over 1,000 *Candidatus* names has been compiled^21^ and recently 917 *Candidatus* names were published as part of a study of the chicken fecal microbiome^22^. The SeqCode was deliberately developed with very few requirements in the effective publication to allow these and other names to be validated (Table 2). In fact, any *Candidatus* name in the literature can be validated under path 2 as long as the taxa are named in the effective publication and a genome meets data quality standards required of the nomenclatural type (see Supplementary Information for an example). This is possible because critical data, including designation of the nomenclatural type, can be captured in the SeqCode Registry during validation. We plan to initiate this effort, which will be done in collaboration with the community. However, the authors of *Candidatus* taxa themselves are welcome to validate names that are already effectively published and meet the sequence quality standards. Because the SeqCode Registry is already operational, this could begin immediately. The basic procedure to validate large numbers of *Candidatus* names is: (1) assess genome sequences assigned to each *Candidatus* taxon for data quality; (2) where a sequence is of sufficient quality to serve as a type, contact authors to check autofilled templates generated by the SeqCode Registry and complete missing data fields; (3) complete validation in the SeqCode Registry; and (4) publish a paper with collaborators from the community announcing validation of the names. This project would result in validation of *Candidatus* names, centralize names and metadata for these taxa, serve an important outreach function to educate the community about the principles and implementation of the SeqCode and provide a conduit for community feedback.

### Data standards

Table 3 summarizes the SeqCode Organizing Committee’s recommendations on minimal standards for data and reporting requirements. These standards were chosen to enable the accurate delineation of species^1,23^ and incorporated many of the recommendations of the Genomic Standards Consortium^16,24^. The SeqCode Organizing Committee discussed the criteria for the original publication of new names using DNA sequences as type at length. The majority felt that the publication requirements should enable the naming of all scientifically well-supported names. For instance, it is not necessary to require the genome accession number in the publication because it will be readily available in the SeqCode Registry. This will allow post hoc registration of *Candidatus* names where the type genomes may not have been explicitly identified. However, it is highly recommended that publications in the future contain the accession number. Similarly, whether the 16S ribosomal RNA sequence should be required or recommended was discussed. The majority opinion was that the 16S rRNA sequence is not necessary for the diagnosis of species and it should not be required. Nevertheless, the entire Committee recognized that the modern taxonomy of prokaryotes is based on the phylogeny of the 16S rRNA and inclusion of an accurate 16S rRNA sequence provides access to this taxonomy as well as an enormous database of environmental ribotypes. For those reasons, the inclusion of an accurate 16S rRNA sequence is highly recommended, although we recognize that rRNA genes can be difficult to assemble and bin accurately because they are often present in multiple copies and do not conform to nucleotide word frequency patterns of coding sequences. While outside the code itself, these standards are in an appendix to the SeqCode and should generally be applied unless there is a strong justification for validating names with lower quality genomes as types (for example, medium-quality genomes with large datasets on physiology, ecology or evolution). We expect that these standards will evolve to keep pace with community feedback and methodological improvements.

While the SeqCode itself is necessarily comprehensive, we have also developed resources to guide the community, including a glossary and examples of the types of data for naming (Supplementary Information).

## Discussion

One goal of the SeqCode is to reverse the trend wherein taxonomic names are published in the primary literature but not validly published. Although the community is free to publish taxonomic names that do not comply with codes of nomenclature, we argue that codes of nomenclature and taxonomic frameworks serve the greater community by promoting objectivity, best practices, communication and data interoperability. However, the unique restrictions of the ICNP regarding viable and accessible type strains have alienated many microbiologists and engendered a sense of normalcy in publishing names outside of the regulation of the ICNP. The SeqCode addresses this problem by providing an efficient and user-friendly resource that serves the common interests of the wider research community. The SeqCode embraces findability, accessibility, interoperability and reusability (FAIR) principles and the Registry was developed with interoperable data structures to promote sharing of SeqCode names across global biodiversity inventories within microbiology and the broader biology research communities (for example, NCBI^25^, GTDB^26^, MiGA^27^, LPSN^28^, Catalogue of Life^29^ and Global Biodiversity Information Facility^30^).

In closing, we emphasize a few important points. First, the SeqCode is not intended to discourage cultivation. Cultivation of mixed or pure cultures enables testing of properties predicted from genomes under controlled conditions. Furthermore, investigators are strongly encouraged to deposit strains to culture collections to improve strain availability, enable assessment of reproducibility of phenotypic traits, provide resources for biochemistry and biotechnology and promote international cooperation. Second, like all other codes of nomenclature, the SeqCode does not provide rules or recommendations on the delineation of taxa. Existing and improving approaches and data structures are available for that purpose^26,27^ and proposals for description of previously undescribed taxa must be settled through peer review. Finally, this is the first version of the SeqCode and we hope that it will evolve as the community engages in further development of the system. Because of our desire to serve the broad microbiology research community, we will engage the community to gather feedback and develop bylaws for SeqCode administration. This code is driven by bottom-up desires to improve communication across the microbial sciences. Thus, we view this ‘SeqCode v.1.0’ as a necessary first step toward a unified system of nomenclature to communicate the full diversity of prokaryotes and we will cooperate with the community toward the realization of this vision.

## Methods

### Public outreach and consensus building

Over the course of the project, considerable effort was spent to communicate with the research community to build consensus on the path forward in microbial systematics. To obtain consensus, four major workshop series were held. The first outreach effort was a three-part web workshop series entitled ‘Microbial systematics for the next decade’, which was held in October 2018. The workshops were intended to engage a diversity of stakeholders in discussions about key issues that affect the landscape of microbial systematics. Each workshop included two 15 min presentations, followed by 15 min of discussion in breakout groups of four to five participants and 15 min of reporting by the breakout groups. Postworkshop questionnaires captured responses to general questions about the future direction of prokaryotic systematics. To maximize productivity, all participants were given reading assignments and asked to develop opinions and ideas for discussions before each seminar. To ensure broad viewpoints, speakers included experts in microbial systematics and from the related fields of plant and protozoal taxonomy. The three workshop themes were as follows. (1) What’s in a name? The importance (and limitations) of formal codes of taxonomic nomenclature. (2) *Candidatus* status: current system and proposed modifications. (3) Efforts to scale and systematize taxonomy in the twenty-first century. Thirty-nine participants from four continents contributed to this first workshop series, which provided a strong foundation for the more decisive and more inclusive workshops to come.

This initial workshop was followed by two in-person workshops. At the first, 28–31 October 2018, in Hood River, Oregon, United States, 24 participants used poll responses from the first set of workshops to narrow in on major issues in microbial systematics and possible solutions. Following plenary presentations and discussions, breakout groups focused on: (1) microbial systematics within a broader perspective; (2) current proposals on the nomenclature of SAGs and MAGs (DNA as a category of nomenclatural type, granting priority to *Candidatus* names, erecting a parallel system of nomenclature or no action); (3) the genomic tree of life; and (4) microbial nomenclature—progressivism versus conservatism. This workshop, in addition to the initial online series led to a consensus statement^2^ proposing two possible paths forward, ‘plan A’, amendment of the ICNP to allow DNA sequence data to serve as a category of nomenclatural type or, pending failure of ‘plan A’, the alternative ‘plan B’, entailing development of a new code of nomenclature based on DNA sequence data as the unifying category of nomenclatural type for cultivated and uncultivated prokaryotes.

The second in-person workshop was held on 8–9 April 2019, in Walnut Creek, California, United States with 27 participants. It focused on scalability and database development related to microbial nomenclature under the two possible plans resulting from the previous workshops. The location and timing of this workshop was coordinated with the US Department of Energy’s Joint Genome Institute (JGI) ‘Genomics of energy and environment meeting’ to take advantage of strong database and bioinformatics expertise available at the JGI and among attendees. Major questions that were a focus of the workshop were as follows. (1) What are the most pressing taxonomic database issues that can help launch microbial taxonomy into the next decade? (2) Is there a way to reach a consensus for a common nomenclature or taxonomy that is treated equally or cross-referenced faithfully in multiple databases? (3) Is there way to facilitate data-rich systematics in the future? This workshop, combined with the subsequent negative vote on previous proposals to amend the ICNP to include DNA sequence data as an alternative category of nomenclatural type^8^ (‘plan A’), finally triggered the writing of the first draft the SeqCode (‘plan B’).

In lieu of a session and subsequent in-person workshop at the ISME18 conference originally scheduled in Cape Town in 2020, which was cancelled due to the COVID19 pandemic, a last series of online workshops was held in February 2021 (SeqCode Workshops, ISME (https://www.isme-microbes.org)). These workshops centred around the first complete draft of the SeqCode, which was shared with all participants before the workshop to drive critical review of the document and its underlying principles. This final series comprised two workshops, each of which had two sessions, one timed for the convenience of participants in Europe, Africa and the Americas and one timed for participants from Asia and Oceania. It was cosponsored by the International Society of Microbial Ecology as part of a developing partnership for administration of the SeqCode. The first workshop was entitled the ‘Path forward for naming the uncultivated’ and included six prerecorded lectures introducing the various topics and discussion and breakout sessions. The second workshop was entitled ‘Path forward to implementations and adoption of the SeqCode’ and included 13 presentations on eukaryotic systematics, databases and related topics. The workshops were highly anticipated and attended by a broad group of microbiologists from all over the world, including 848 registrants and at least 575 attendees from 42 countries on 6 continents. Participants identified with a broad range of subdisciplines within microbiology, including microbial ecologists and systematists. These two communities do not often interact and the strong participation of both groups was a strength of the workshops. A total of 26% of respondents identified as graduate students. We note that training for microbial systematics is almost non-existent, despite the large number of scientists using taxonomic names. Thus, career development was a significant outcome of these workshops. A total of 95% of respondents said the content and outcomes of the workshops will be useful to them and/or their field and 90% said they are likely to use SeqCode in the future. Given the strong participation and near-unanimous support for SeqCode, the SeqCode committee incorporated feedback from breakout groups that tackled key questions about the SeqCode, which were carefully considered and acted on by the SeqCode Committee, as summarized in Supplementary Table 1.

### Reporting summary

Further information on research design is available in the Nature Research Reporting Summary linked to this article.

## Supplementary information

Supplementary Information Includes the SeqCode, glossary and examples of nomenclature proposals under the SeqCode.

Reporting Summary

Supplementary Table Summary of breakout group discussions from SeqCode workshops.

## Data Availability

Data sharing not applicable to this article as no datasets were generated or analysed during the current study.

## Code availability

Code and data associated with the SeqCode Registry are accessible and reusable through the Creative Commons Attribution 4.0 License, except where otherwise noted, and the underlying code is released as open source under the terms of the Massachusetts Institute of Technology License.
