# Supplementary material for: SeqCode: a nomenclatural code for prokaryotes described from sequence data
Source: Nat Microbiol. 2022 Sep 19;7(10):1702–8. doi: 10.1038/s41564-022-01214-9 (PMC9519449; doi:10.1038/s41564-022-01214-9)
Supplement: Supplementary file 1 — Includes the SeqCode, glossary and examples of nomenclature proposals under the SeqCode. [file 41564_2022_1214_MOESM1_ESM.pdf]

nature portfolio

https://doi.org/10.1038/s41564-022-01214-9

Supplementary information

SeqCode: a nomenclatural code for prokaryotes described from sequence data

In the format provided by the authors and unedited

SeqCode, a nomenclatural code for prokaryotes described from sequence data

Brian P. Hedlund, Maria Chuvochina, Philip Hugenholtz, Konstantinos T. Konstantinidis, Alison E. Murray, Marike Palmer, Donovan H. Parks, Alexander J. Probst, Anna-Louise Reysenbach, Luis M. Rodriguez-R, Ramon Rossello-Mora, Iain C. Sutcliffe, Stephanus N. Venter, and William B. Whitman*

* Correspondence to: William B. Whitman, whitman@uga.edu

Supplementary Information — Page

The Code of Nomenclature of Prokaryotes Described from Sequence Data 2
Glossary 21
Example cases of validation of names under the SeqCode 23
Example of path 1: family *Wolframiiraptoraceae* and child taxa 23
Table S1: Example spreadsheet format for nomenclature proposals 28
SeqCode Pre-registration 28
Example of path 2: *Macondimonas diazotrophica* 30
Example of combination of paths 1 and 2: class *Kryptonia* and child taxa 32
Table S2. Etymology and typification of higher taxa for *Kryptonium thompsonii* 34
References 36

The Code of Nomenclature of Prokaryotes Described from Sequence Data

Edited by William B Whitman[a], Maria Chuvochina[b], Brian P Hedlund[c], Philip Hugenholtz[b], Konstantinos T Konstantinidis[d], Alison E Murray[e], Marike Palmer[c], Donovan H Parks[b], Alexander J Probst[f], Anna-Louise Reysenbach[g], Luis M Rodriguez-R[h], Ramon Rossello-Mora[i], Iain Sutcliffe[j] and Stephanus N Venter[k]

[a] Department of Microbiology, University of Georgia, Athens, GA, USA

[b] The University of Queensland, School of Chemistry and Molecular Biosciences, Australian Centre for Ecogenomics, Australia

[c] School of Life Sciences, University of Nevada, Las Vegas, Las Vegas, NV, USA

[d] School of Civil and Environmental Engineering, Georgia Tech, Atlanta, GA, USA

[e] Division of Earth and Ecosystem Sciences, Desert Research Institute, Reno, NV, USA

[f] Department of Chemistry, Environmental Microbiology and Biotechnology (EMB), Group for Aquatic Microbial Ecology and Centre of Water and Environmental Research (ZWU), University of Duisburg-Essen, Essen, Germany.

[g] Biology Department, Portland State University, Portland, OR, USA

[h] Department of Microbiology and Digital Science Center (DiSC), University of Innsbruck, Innrain 15 / 01-05, Innsbruck 6020, Austria

[i] Marine Microbiology Group, Department of Animal and Microbial Diversity, Mediterranean Institute of Advanced Studies (CSIC-UIB), Esporles, Illes Balears, Spain

[j] Faculty of Health & Life Sciences, Northumbria University, Newcastle upon Tyne, UK

[k] Department of Biochemistry, Genetics and Microbiology, Forestry and Agricultural Biotechnology Institute (FABI), University of Pretoria, Pretoria, South Africa

CHAPTER 1. GENERAL CONSIDERATIONS

General Consideration 1
The progress of prokaryotic microbiology requires a precise system of nomenclature accepted by the majority of microbiologists. For practical purposes, the term prokaryotes is a synonym for *Archaea* and *Bacteria*.

General Consideration 2
To achieve order in nomenclature, it is essential that scientific names be regulated by internationally accepted rules.

General Consideration 3
The rules that govern the scientific nomenclature used in the biological sciences are embodied in codes of nomenclature.

General Consideration 4
Rules of nomenclature do not govern the delimitation of taxa or determine their relations. The rules are primarily for assessing the correctness of the names applied to defined taxa; they also prescribe procedures for creating and proposing new names.

General Consideration 5
The Code of Nomenclature of Prokaryotes Described from Sequence Data applies to the naming of all prokaryotes where the lower taxa (species and subspecies) are typified by a DNA sequence. This code is colloquially referred to as the SeqCode to distinguish it from the International Code of Nomenclature of Prokaryotes (ICNP), which applies to the naming of all prokaryotes where the lower taxa (species and subspecies) are typified by either a strain or illustration/description. The nomenclature of eukaryotic microbial groups is provided for by other Codes: fungi and algae by the International Code of Nomenclature for algae, fungi and plants; protozoa by the International Code of Zoological Nomenclature. The nomenclature of viruses is provided for by the International Code of Virus Classification and Nomenclature.

General Consideration 6
The Committee on the Systematics of Prokaryotes Described from Sequence Data, colloquially the SeqCode Committee, has been established to provide mechanisms to emend, interpret, and consider exemptions to the rules of the SeqCode.

General Consideration 7
The SeqCode is divided into principles, rules and recommendations. The principles (Chapter 2) form the basis of the Code, and the rules and recommendations are derived from them. The rules (Chapter 3) are designed to implement the principles. The recommendations (Chapter 3) supplement some of the rules and do not have the force of rules. They are intended as guides to desirable practice. Names contrary to recommendations cannot be rejected for this reason. Appendices may be added to assist in the application of this Code and do not form the legislative part of this Code.

General Consideration 8
Nomenclature deals with the application of names to the following taxonomic ranks, i.e., “subspecies”, “species”, “genus”, “family”, “order”, “class”, and “phylum”.

General Consideration 9
The SeqCode is an instrument of scientific communication.

CHAPTER 2. PRINCIPLES

Principle 1
The SeqCode has one fundamental aim, which is to provide a standardized, robust, and stable system of nomenclature for prokaryotes that is compatible with the freedom of scientists to classify prokaryotes according to taxonomic opinion. Nothing in the SeqCode may be construed to restrict the freedom of taxonomic opinion or action.

Principle 2
The nomenclature of prokaryotes is not independent of botanical, zoological and viral nomenclature. When naming new taxa at the rank of genus or higher, names that are already regulated by the International Code of Zoological Nomenclature, the International Code of Nomenclature for algae, fungi and plants and the International Code of Virus Classification and Nomenclature must not be used.

The names formed under the SeqCode are not independent of the names regulated by the International Code of Nomenclature of Prokaryotes (ICNP) and formed before January 1, 2022. Before that date, legitimate names formed under the ICNP have priority. After that date, the rules of priority of the SeqCode are used to determine priority of names formed under the ICNP compared with names formed under the SeqCode.

Principle 3
The scientific names of all taxa are Latin or Latinized words treated as Latin.

Principle 4
The primary purpose of giving a name to a taxon is to supply a means of referring to it. A secondary consideration is that names should aid memorability.

Principle 5
Names of taxa are tied to their nomenclatural types, referred to as types in the SeqCode. Types should serve as a reference point that allow the unambiguous identification of taxa.

Principle 6
The correct name of a taxon is based upon valid publication, legitimacy, taxonomic position, and priority of publication.

Principle 7
A name only has standing in nomenclature if it is validly published under the rules of the SeqCode.

Principle 8

Each taxon with a given circumscription, position, and rank can bear only one correct name, i.e., the earliest that is in accordance with the rules of the SeqCode. The circumscription of a taxon is an indication of its limits or the set of biological entities it contains. The position of a taxon or rank is an indication of its relationship to a parent taxon within a taxonomy.

Principle 9

To promote stability, the name of a taxon should not be changed or replaced without sufficient reason based on either further taxonomic studies or the need to rectify a name that is contrary to the rules of SeqCode.

Principle 10

Names created should be clear enough to avoid errors, confusion, or misunderstandings.

CHAPTER 3. RULES OF NOMENCLATURE WITH RECOMMENDATIONS

Section 1. General

Rule 1

The Code on the Nomenclature of Prokaryotes Described from Sequence Data, or SeqCode, will take effect on January 1, 2022.

Rule 2

The SeqCode Legislative Commission has been established as the legislative branch of the SeqCode Committee in accordance with its statutes. The SeqCode Legislative Commission is the only body authorized to amend the SeqCode.

Rule 3

The SeqCode Reconciliation Commission has been established as the judicial branch of the SeqCode Committee to make decisions pertaining to the application of the SeqCode in accordance with its statutes. Examples of cases for the Reconciliation Commission may include: (a) cases in which the consequences or interpretation of a rule are uncertain, (b) cases in which the application of a name is likely to endanger health or have serious economic consequences, or (c) cases where the application of a rule is likely to lead to confusion. When the opinion of the SeqCode Reconciliation Commission is sought, a summary of pertinent facts should be submitted to the Reconciliation Commission. The SeqCode Reconciliation Commission is the only body authorized to render decisions on the application of the SeqCode.

Rule 4

The SeqCode Registry has been established to record and maintain names that are formed or recognized under the SeqCode in accordance with its statutes. Registration of names constitutes valid publication and is required for naming under the SeqCode.

Rule 5
The rules of the SeqCode are retroactive except where specified.

Rule 6
Names contrary to a rule may not be maintained in the SeqCode Registry.

Section 2. Ranks of Taxa

Rule 7a
The taxonomic categories covered by these rules are given below in descending taxonomic rank.

Phylum
Class
Order
Family
Genus
Species
Subspecies

Rule 7b
The relative order of these categories may not be altered in any classification even though definitions of taxonomic categories may vary with individual opinion.

Rule 7c
The use of the taxonomic category of subspecies is optional.

Rule 7d
Intermediate or informal ranks or categories not mentioned in Rule 7a are not covered by the SeqCode.

Recommendation 7: An author who describes and names a new taxon should indicate the rank of the taxon and where possible the rank and name of the parent taxon. If the parent taxon is not currently named, the author should name it.

Section 3. Naming of Taxa

General

Rule 8
The scientific names of all taxa must be treated as Latin and spelled only with the Latin alphabet. A species name is a binary combination of a genus name and a specific epithet; names of taxa above the rank of species are single words. Typographical signs, numbers, and additional characters cannot be used.

Rule 9a

A name at any taxonomic rank can only refer to a single type.

Rule 9b

A later homonym of a name formed under the nomenclatural codes listed in Principle 2 cannot be used.

Recommendation 9

To form new prokaryotic names, authors are advised as follows:

1. Names that are very long or difficult to pronounce should be avoided.
2. Names should differ by at least three characters from existing names of genera or species within the same genus.
3. Languages other than Latin should be avoided when Latin equivalents exist or can be constructed by combining Latin word elements. Exceptions include names derived from local items such as foods, drinks, geographic localities, and other names for which no Latin words exist.
4. Authors should not name organisms after themselves. If names are formed from personal names, they should contain only the name of one person. They may contain the untruncated family and/or first names.
5. All personal genus names should be feminine regardless of the gender identity of the person they commemorate.
6. Names should not be deliberately contentious or abusive of any person, gender or sexual identity, race, religion, political belief, or ideology.
7. Names that include mnemonic cues are preferred because they promote learning and memory.

Names of Genera

Rule 10

The name of a genus is a noun or adjective used as a noun, in the singular number and written with an initial capital letter.

Recommendation 10

Authors should attend to the following Recommendation and those of Recommendation 9 when forming genus names.

1. Names that have the same suffixes as those used for the higher taxonomic ranks should be avoided: -*aceae*, -*ales*, -*ia*, and -*ota* (see Table 1).

Names of Species

Rule 11

The name of a species is a binary combination consisting of the name of the genus followed by a single word known as a species epithet. The genus part of the name must begin with an initial capital letter, and the species epithet must begin with a lowercase letter.

Rule 12

A species epithet must be related to the genus name in one of three ways.

1. As an adjective. Example: *aureus* in *Staphylococcus aureus*.
2. As a substantive (noun) in apposition in the nominative case. Example: *Desulfovibrio gigas*https://doi.org/10.1601/nm.3486 or other names cited in Trüper and De'Clari (1997).
3. As a noun in the genitive case. Example: *coli* in *Escherichia coli*.

Recommendation 12

Authors should attend to the following recommendations and those of Recommendation 9 when forming species names.

1. When a species epithet is chosen to indicate a property or source of the species, epithets should not express a character common to all, or nearly all, the species of a genus.
2. When the species epithet is an adjective, it should agree in gender with the genus name.

Names of Subspecies

Rule 13a

The name of a subspecies is a ternary combination consisting of three names: the genus name, the species epithet, the abbreviation "subsp." (subspecies), and the subspecies epithet that begins with a lower-case letter.

Rule 13b

A subspecies epithet is formed in the same way as a species epithet.

Rule 13c

A subspecies that includes the type of the species must bear the same epithet as the species.

Names of Taxa above the Rank of Genus (Family, Order, Class, Phylum)

Rule 14

The name of a taxon above the rank of genus is a Latinized word. Names of families and orders are in the feminine gender, the plural number, and written with an initial capital letter. Names of classes and phyla are in the neuter gender, the plural number, and written with an initial capital letter.

Rule 15

The name of a family, order, class, or phylum is formed by the addition of the appropriate suffix to the stem of the type genus name (see Section 4). These suffixes are presented in Table 1.

Table 1. Suffixes for Taxonomic Ranks

Rank	Suffix	Example for the genus *Hadarchaeum*[a]
Phylum	*-ota*	*Hadarchaeota*
Class	*-ia*	*Hadarchaeia*
Order	*-ales*	*Hadarchaeales*
Family	*-aceae*	*Hadarchaeaceae*

[a]From Chuvochina et al., 2019

Section 4. Nomenclatural Types and Their Designation

General

Rule 16

Each named taxon must have a designated nomenclatural type. The nomenclatural type, referred to in the SeqCode as "type", for a species or subspecies is the evidence for that taxon (DNA sequence, see Rule 18a) with which the name is permanently associated. For taxa above the rank of species, the type is one of the subordinate taxa, with which the name is permanently associated. Formation of names of the taxa above the level of genus is based on the names of the types, allowing tracing which biological entity is included in the taxon. The nomenclatural type is not necessarily the most typical or representative element of the taxon.

Types of the various taxonomic categories are presented in Table 2.

Table 2. Nomenclatural types of taxonomic ranks

Taxonomic category	Nomenclatural type
Subspecies	Designated DNA sequence
Species	Designated DNA sequence
Genus	Designated species
Family	Designated genus
Order	Designated genus
Class	Designated genus
Phylum	Designated genus

Rule 17

The type of a taxon must be designated for the name to be validly published (see Section 5).

Type of a Species or Subspecies

Rule 18a

The type of a species or subspecies is a designated DNA sequence that is compliant with the minimum standards designated by the SeqCode Committee for genome, metagenome-assembled genome, or single-amplified genome sequences. The sequence must be available in the International Nucleotide Sequence Database Collaboration (INSDC). Upon recommendations of

the SeqCode Committee or subcommittees on the taxonomy of specific groups, the SeqCode Committee may approve other minimal standards as suitable types for specific groups.

Rule 18b

The type of a species or subspecies must allow the unambiguous identification of the taxon. Names based on types that later prove to be ambiguous are not legitimate unless a neotype is proposed.

Rule 18c

If the type of a name is lost or demonstrated to be ambiguous, a neotype sequence may be proposed to the SeqCode Reconciliation Commission. If approved, the SeqCode Registry will be amended to reflect the new type.

Rule 19

Unless designated under the rules of this code, a reference DNA sequence is not a type but a sequence used in comparative studies. A reference sequence has no standing in nomenclature.

Recommendation 19

When a strain belonging to a taxon named under the SeqCode is isolated, a reference strain should be designated and submitted to two culture collections in different countries. Reference strains have no standing in nomenclature.

Types of higher taxa

Rule 20

Only taxa with legitimate names may serve as types for taxa higher than the rank of species.

Rule 21a

The nomenclatural type of a genus is the type species that was designated when the genus name was originally validly published.

Rule 21b

The valid publication of a new genus name as a deliberate substitute for an earlier name found to be illegitimate does not change the type species of the genus.

Rule 22

When more than one subordinate taxa are available to serve as type, the earliest legitimately named taxon available at the time must be chosen, except where the type is neither a strain nor sequence data (i.e., taxa described from illustrations under the ICNP).

Section 5. Priority and Valid Publication of Names

Rule 23a

Any taxon with a given circumscription, position, and rank can bear only one correct name, the earliest name that is in accordance with the rules of SeqCode.

Note 1. In the case of a species epithet, Rule 23a must be applied independently of the genus name. Under most circumstances, the species epithet remains the same on transfer of a species from one genus to another. However, if the species epithet is currently in use in the name of another species or subspecies in the genus to which the species is to be transferred, a new name must be proposed for the transferred species.

Note 2. In the case of a subspecies, Rule 23a must be applied independently of the genus name and species epithet. The subspecies epithet remains the same on transfer of a subspecies from one species to another unless the subspecies epithet has been previously used as the name of another species or subspecies in the genus to which the subspecies is to be transferred.

Rule 23b
The priority of a genus, species, or subspecies name is determined by the time and date of its valid publication, i.e., when the registration of the name is completed. For purposes of priority, only legitimate names are taken into consideration.

Rule 23c
The priority of species and subspecies names will compete for priority with names in any other code after January 1, 2022. If two names validly published after 1 January 2022 compete for priority, priority is determined by the time and date of valid publication, either under the rules of the SeqCode or other nomenclatural codes. If both names are published at the same time and date, priority will be decided by the SeqCode Reconciliation Committee.

Rule 23d
The priority date of names of taxa of rank higher than genus proposed after 1 January 2022 is the same as the priority date of the corresponding type genus name. The priority date for names published before 1 January 2022 is the same as their priority under the ICNP.

Rule 23e
Legitimate names validly published under the ICNP remain legitimate in the SeqCode even if there are differences in type designations.

Effective publication

Rule 24a
Effective publication under the SeqCode means that the name and evidence for the taxon have been published in a peer-reviewed journal or book.

Rule 24b
When a name of a new taxon is published in a work written in a language other than English, the author(s) should include a description in English in the publication.

Rule 24c

The following are not accepted as forms of effective publication.

1. Communication of new names at a meeting, minutes of a meeting, or abstracts of papers presented at meetings.
2. Placing of names in listings or catalogues of collections.
3. Reports in ephemeral publications, newsletters, white papers, self-published papers, or non-scientific periodicals.
4. A published patent application or issued patent including the name.
5. A database containing names associated with a sequence or metadata.
6. Electronic material available in advance of publication (e.g., papers in press or preprints).

Valid and Invalid Publication and Registration of Names

Rule 25

The time and date of validation is the time and date of completion of the registration in the SeqCode Registry.

Rule 26

A name of a new taxon, or a new combination for an existing taxon, is not validly published unless the following criteria are met:

1. The name is effectively published under the rules of the SeqCode.
2. The name is registered in the SeqCode Registry, along with mandatory data fields listed below.
3. The type of the taxon is clearly designated. In the case of species or subspecies, the type sequence is deposited according to Rule 18a and the accession number cited.
4. The taxonomic rank is designated.
5. The derivation (etymology) of a new name (and if necessary of a new combination) is given, wherein one or more distinguishable roots are identified. Roots can originate from any language in use or extinct (see also Recommendation 9).

Note 1. When a new species or a new combination results in the proposal of a new genus, both the new genus name and the new species name or the new combination must be validly published. Publication of the new species epithet or new combination alone does not constitute valid publication of the new genus name.

Note 2. When possible, authors are recommended to include the SeqCode Registry identifier in the effective publication.

Note 3. If the information provided in the registration and the effective publication differ, the registration is considered definitive.

Recommendation 26

It is recommended that the name, etymology, type information, and diagnosis of the novel taxon should be clearly identifiable in a designated section of the effective publication (i.e., the section termed the ‘protologue’ by some microbial taxonomists). Authors are encouraged to provide

information describing the taxon, such as predicted or known physiological characteristics, ecological data, location, and additional metadata. Authors are also encouraged to submit metadata with the type sequence in one of the INSDC databases.

Rule 27

Placement of a species or subspecies epithet into a genus or species that is illegitimate does not preclude the legitimacy of the species or subspecies epithet.

Section 6. Citation of Authors and Names

Proposal and Subsequent Citation of the Name of a New Taxon

Recommendation 28

The effective publication should be cited with the name of a previously proposed taxon. Correct citation of a name enables the date of publication, the description, and the circumscription of the taxon to be found. For names published under the SeqCode, the validly published name and date of valid publication should be determined from the SeqCode Registry.

Proposal of a New Combination

Rule 29

When an author proposes transfer of a species to another genus, or a subspecies to another species, then the author who makes the proposal should indicate the formation of the new combination by the addition of the abbreviation ‘‘*comb. nov*.’’ (*combinatio nova*). This convention should be used when the author retains the original species epithet in the new combination. However, if an author is obliged to substitute a new species epithet as a result of homonymy, the abbreviation ‘‘*nom. nov.*’’ (*nomen novum*) should be used. The original name is referred to as the basonym and should be given, along with the citation of the effective publication, in the description of the novel combination.

Citation of the Name of a Taxon whose Circumscription Has Been Emended

Recommendation 30

If an alteration of a taxon modifies its circumscription, the author responsible may be indicated by the addition to the author citation of the abbreviation ‘‘*emend.*’’ (*emendavit*) followed by the name of the author responsible for the change. Only alterations that cause significant changes in the circumscription warrant description as an emendation.

Section 7. Changes in Names of Taxa as a Result of Transference, Union, or Change in Rank

Rule 31

If the type of a taxon is excluded, a type must be designated for the remaining members of the original taxon (see Rule 17), which must be given a new name.

Rule 32

A change in the name of a taxon is not warranted by an alteration of the diagnostic characters or the circumscription.

Rule 33

If a genus is divided into two or more genera, the genus name must be retained for the genus that retains the type species.

Division of Species and Subspecies

Rule 34a

When a species is divided into two or more species, the species epithet of the original species must be retained for the taxon that includes the type.

Rule 34b

When a species is divided into two or more subspecies, the species epithet of the original species must be retained for the subspecies that includes the type.

Note. Although the species and subspecies epithets in the name of a type subspecies are the same, they do not contravene Rule 9 because they are based on the same type.

Rule 34c

When a subspecies is divided into two or more subspecies, the subspecies epithet of the original subspecies must be retained for the subspecies that includes the type.

Transfer of a Species to Another Genus

Rule 35a

When a species is transferred to another genus without any change of rank, the species epithet must be retained unless it is already in use in the new genus. In that case, a new species epithet must be chosen for the transferred species. This rule avoids creation of a later homonym.

Rule 35b

Regardless of its priority, the transfer of species that is not the type of another genus does not affect the type species or priority of the receiving genus even if it involves union of the incoming species with the type species of the receiving genus.

Rule 35c

When the name of a genus is changed, the epithets of the species within it must be retained unless already in use (see Rule 29).

Note 1: Modification of the gender of the species epithet to accommodate the gender of the new genus name is a minor orthographic variant and encouraged.

Union of Taxa of Equal Rank

Rule 36
When two or more taxa of the same rank are united, then the name and type of the taxon is determined by following the rules of priority (see Section 5). In cases of species and subspecies, if the names or epithets are of the same time and date, the author who first unites the taxa has the right to choose one of them, and their choice must be followed.

Rule 37
When several species are united under one species as subspecies, the subspecies that includes the type of the species under whose name they are united must be designated by the same epithet as the species name (see Rule 13c).

Rule 38
If two or more species of different genera are united to form a single genus, and if those species include the type species of one or more genera, the genus name must be the earliest validly published, legitimate name. If no type species is placed in the genus, a new genus name must be proposed, and a type species must be specified.

Rule 39
When two or more taxa of the same rank from family to class are brought together under a taxon of higher rank, the higher-ranking taxon should derive its name from the name of the earliest legitimate type genus among the lower-ranking taxa.

Rule 40
If no type genera were placed in the taxon, a new name based on the selected type must be proposed. (see Rule 22)

Change in Rank

Rule 41a
When the rank of a taxon of genus or above is changed, the stem of the name must be retained and only the suffix altered (see Rule 15).

Rule 41b
When a subspecies is elevated in rank to a species, the subspecies epithet must be used as the species epithet unless the resulting combination is illegitimate.

Rule 41c
When a species is lowered in rank to a subspecies, the species epithet must be used as the subspecies epithet unless the resulting combination is illegitimate.

Section 8. Illegitimate Names and Epithets: Replacement, Rejection, and Conservation of Names and Epithets

Illegitimate Names

Rule 42
A name contrary to a rule is illegitimate and must not be used. However, a name of a taxon that is illegitimate when the taxon is in one taxonomic position is not necessarily illegitimate when the taxon is in another taxonomic position.

Note: Some common reasons for which a name may be illegitimate are the following.

1. If the taxon to which the name was applied, as circumscribed by the author, included the nomenclatural type of a name which the author ought to have adopted under one or more of the rules.
2. If the author did not adopt for a binary or ternary combination the earliest legitimate genus name, species epithet, or subspecies epithet available for the taxon with its particular circumscription, position, and rank.
3. A species or subspecies epithet is illegitimate if it duplicates a species or subspecies epithet previously validly published for the same genus but whose name is based upon another type.

Replacement of Names

Rule 43
An illegitimate name or epithet should be replaced by the earliest legitimate name or epithet in a binary or ternary combination which in the new position will be in accordance with the rules. If no legitimate name or epithet exists, one must be chosen. Since a species epithet is not rendered illegitimate by publication in a species name in which the generic name is illegitimate, authors may use such an epithet if they wish provided that there is no obstacle to its employment in the new position or sense; the resultant combination is treated as a new name *(nom. nov.)* and is ascribed to the author. The epithet is, however, ascribed to the original author.

Rule 44
A legitimate name or epithet may not be replaced.

Rule 45
Names contrary to the General Considerations or Principles of the code may be rejected by the SeqCode Reconciliation Commission.

Section 9. Orthography

Rule 46

All names comprise only the 26 letters of the ISO basic Latin alphabet. Diacritic signs are not to be used.

Rule 47

Any name or epithet should be written in conformity with the spelling of the word from which it is derived and in accordance with the rules of Latin grammar. Exceptions are provided for typographic and orthographic errors and orthographic variants.

Notes:

1. Consult Appendix 9 of the ICNP for recommendations on forming properly Latinized names.

2. In the SeqCode an orthographic variant is a name (or epithet) applied to the same type that differs from another name only in transliteration into Latin of the same word from a language other than Latin or in its grammatical correctness. Changes in suffixes for consistency with the names of higher taxa are orthographic variants. Names transliterated from the same word and based on different types are not orthographic variants.

Typographic and Orthographic Errors

Rule 48

The original spelling of a name or epithet must be retained, except for typographical or orthographic errors.

An unintentional typographical or orthographic error later corrected by the author is to be accepted in its corrected form without affecting the status and date of valid publication. It can also be corrected by a subsequent author who may or may not mention that the spelling is corrected. However, the abbreviation ‘‘corrig.’’ (*corrigendum*) may be appended to the name if an author wishes to draw attention to the correction. Succeeding authors may be unaware that the original usage was incorrect and use the spelling of the original author(s). Other succeeding authors may follow the correction of a previous author or may independently correct the spelling themselves, but in no case is the use of corrig. regarded as obligatory. None of these corrections affects the status and date of validation.

Note. The liberty of correcting a name or epithet must be used with reserve, especially if the change affects the first syllable and above all the first letter of the name or epithet.

Personal Names

Rule 49

The genitive and adjectival forms of a personal name are treated as different epithets and not as orthographic variants unless they are so similar as to cause confusion.

Gender of Names

Rule 50

The gender of genus names is governed by the following.

1. A Latin or Latinized genus name retains the gender of its language of origin. Authors must give the gender of any proposed genus name. In cases where the classical gender varies, the author has the right of choice between the alternatives.
2. Genus names that are compounds from two or more Latin words take the gender of the last component of the compound word.
3. Arbitrarily formed genus names or vernacular names used as genus names take the gender assigned to them by their authors.

CHAPTER FOUR. RECOMMENDATIONS FOR AUTHORS AND PUBLISHERS

When it is desirable to distinguish the nature of the type of a name, the following convention is recommended. When the type for a species or subspecies is determined by the ICNP, the superscript "T" will be used immediately following the name or strain identifier. If the type is determined by the SeqCode, the superscript "Ts" or "TS" will be used. When the type is a taxon at the rank of genus or higher, the superscript is determined by the nature of the type of the species. If superscripts are not possible, they may be replaced by the symbols in parentheses, i.e., (T), (Ts) or (TS).

For the purpose of identification in the text, names of taxa at all ranks should be italicized.

REFERENCES

Chuvochina M, Rinke C, Parks DH, Rappé MS, Tyson GW, Yilmaz P, Whitman, WB, Hugenholtz P (2019) The importance of designating type material for uncultured taxa. Syst Appl Microbiol 42 (2019) 15–21

Trüper HG, de'Clari L (1997) Taxonomic note: necessary correction of specific epithets formed as substantives (nouns) "in Apposition". Int J Syst Bacteriol 47:908–909.

APPENDIX 1

Data quality and reporting requirements and recommendations for an isolate genome, metagenome assembled genome (MAG), or single amplified genome (SAG) to serve as the nomenclatural type for a species named under the SeqCode. Requirements will be checked as part of the validation process on the SeqCode Registry. Recommendations are suggested best practices to guide authors and peer reviewers to ensure high quality data supporting species to be named.

To be included in publication proposing new species names under SeqCode

Required

Name

Recommended

Etymology

Name formed with mnemonic cues

Interpretation of biological properties inferred or demonstrated physiological traits and ecological information, such as habitat, in the manuscript body and/or protologue.

Designated genome assembly (e.g., INSDC accession) and access to raw data (e.g., SRA accession).

Include as much metadata as possible (see Field et al., 2008).

Provide evidence of the species, taxonomic rank, and position including the uniqueness of the species with respect to existing named species and justify the taxonomic rank and position (e.g., Jain et al., 2018, Karthikeyan et al., 2019; Parks et al., 2020; Rodriguez-R et al., 2018).

For MAGs and SAGs, compare multiple high-quality genomes representing the species in more than one sample (e.g., Supplemental Information).[a]

Rationale: Initial requirements encourage wide participation from many microbiological disciplines and enable validation of names published prior to the SeqCode. Critical data will be captured in the SeqCode Registry in any case. Some recommendations could become requirements in the future.

Data quality necessary for completion of SeqCode Registry[b]

Required

Type genome assembly quality for MAGs and SAGs: >90% complete and <5% contaminated; (modified from Bowers et al., 2017).

For isolates, read coverage ≥10x (Field et al., 2008).

Recommended

16S rRNA genes >75% complete and passes chimera checks

Agreement between genome and 16S rRNA taxonomic assignments

>80% of tRNAs present (modified from Bowers et al., 2017).

High genome integrity (contig # <100; N50 >25 kb; max. contig >100 kb).

MAG/SAG read coverage ≥10x.

Data availability required for SeqCode Registry

Assembly available in INSDC databases

	Raw data available in INSDC databases (e.g., Sequence Read Archive)[c] **Rationale:** Registry queries the INSDC databases to perform automatic checks of data quality

a. Comparison of multiple high-quality genomic assemblies from multiple samples can support the non-chimeric nature of MAGs and provide confidence of the assembly for both MAGs and SAGs.
b. Data quality will be assessed by automated pipelines or other approaches. Exceptions for lower data quality should be justified by authors in the effective publication.
c. Not required for names effectively published before January 1, 2023, to allow for existing published names (e.g., existing *Candidatus* names) and names currently undergoing peer review to be validated under the SeqCode.

Glossary

Basonym: The original or earliest validly published name of a taxon on which a new combination is based when this taxon has been changed, typically as a result of reclassification.

comb. nov. (*combinatio nova*): Abbreviation to denote transfer of a species to a new genus. Because the species name includes both the genus name and species epithet, the new name is a combination of the new genus name and the original species epithet. See Rule 29.

Correct: Validly published names or epithets that must be adopted for a taxon under the rules in the proposed taxonomy (i.e., circumscription, position and rank). Note that validly published names may be legitimate but not correct, depending on the taxonomy. For instance, a given taxonomy may merge two species. While both names are validly published and legitimate, only the name of the earlier synonym is correct in this taxonomy.

Diagnosis: A statement of the characters that allows one to distinguish the taxon from other taxa.

Earlier synonym: The first validly published synonym [in some taxonomic literature, this is referred to as senior synonym].

Epithet: An adjective or descriptive phrase expressing a quality characteristic of the person or thing mentioned. The species name is an epithet of the genus name.

Effectively published: Names and epithets that have appeared in print and/or electronic matter that is peer reviewed and made generally available to the scientific community. Under the SeqCode, preprints are not eligible as an effective publication. See Rule 24 for more explanation.

Effective publication: The peer-reviewed publication in which a name first appears. Under the SeqCode, preprints are not eligible as an effective publication. See Rule 24 for more explanation.

Homotypic synonyms: Two or more names associated with the same type [in some taxonomic literature, these are referred to as objective synonyms].

Heterotypic synonyms: Two or more names associated with different types that in the opinion of the microbiologist concerned belong to the same taxon [in some taxonomic literature, these are referred to as subjective synonyms].

Homonymy: A situation in which two names are the same but have different types, often due to uniting two genera each of which contains species with the same species epithet or when a name is chosen that is already in use by another taxon, including taxa named under other international codes of nomenclature.

Illegitimate: Names or epithets that are contrary to the rules of the SeqCode.

Later synonym: A subsequently validly published synonym [in some taxonomic literature, this is referred to as junior synonym].

Legitimate (names and epithets): In accordance with the rules of the SeqCode.

Name: The label applied to an object. A word or set of words by which a taxon is known, addressed, or referred to. A term used for identification.

nom. nov. (*nomen novum*): A name that is created to replace another scientific name in cases where an earlier name cannot be used for technical, nomenclatural reasons. See Rules 29 and 43.

Nomenclature: The body or system of names in a particular field.

Nomenclatural type (or just type): For a species, it is the evidence for the taxon, such as genome sequence, with which the name is permanently associated. In the ICNP, the type is a viable strain deposited in and available from two culture collections in different countries. For higher taxa, the type is a lower taxon. The nomenclatural type is not necessarily the most typical or representative element of the taxon.

Orthography: Formation of words or names.

Orthographic variant: Names or epithets that are variant spellings of the same name. Under the SeqCode, orthographic errors may be corrected without affecting the priority of the name. See Rules 46 and 47.

Position: The higher taxon in which a taxon is placed when there may be alternatives.

Principle of Priority: A principle that states that the correct name of a taxon is the oldest legitimate, validly published name applied to it. It is the fundamental guiding precept that preserves the stability of biological nomenclature. It was first formulated in 1842 by a committee appointed by the British Association to consider the rules of zoological nomenclature. The committee's report was written by Hugh Edwin Strickland.

Rank: A level within a taxonomic hierarchy. In the SeqCode, ranks are restricted to phylum, class, order, family, genus, species, and subspecies. See Rule 7.

Species name: A binomial combination of the genus name and species epithet. See Rules 11 and 12.

Taxon: Any group of organisms treated as a named group in a formal taxonomy.

Validation (of a name or epithet): The process by which a name becomes "official" under a particular code of nomenclature. Under the SeqCode, validation is completed when the registration process is complete on the SeqCode Registry. See Rule 26.

Validly published: Names and epithets that are effectively published and registered according to the rules of the code. See Rule 26.

Examples of application of the SeqCode

Example of path 1: *Wolframiiraptoraceae* and child taxa

Overview. As an example case for Path 1, several authors of the SeqCode (Palmer, Reysenbach, Hedlund) recently completed a combined cultivation/metagenomics study of a novel group of archaea in the GTDB family designated as NZ13-MGT within the GTDB order "Caldarchaeales", class *Nitrososphaeria*, and phylum *Thermoproteota*. The study initially focused on anaerobic enrichment cultures from sediments of Great Boiling Spring, Nevada, USA, with a single member of the taxon that requires tungsten for growth on corn stover or a sugar mix. The taxon was shown to prefer xylose by combining fluorescence *in situ* hybridization (FISH) with nanometer-scale secondary ion mass spectrometry (nanoSIMS). It is currently represented by a single high-quality MAG, although that MAG formed a >99.5% average nucleotide identity (ANI) cluster with MAGs of lower quality from separate samples of the same enrichment culture and sediments from which the enrichment culture was derived (Buessecker et al., 2022). Analysis of the MAG revealed a putative TupA tungstate transporter and six annotated tungsten-dependent ferredoxin oxidoreductases. To expand the study, 77 additional high-quality MAGs assigned to the GTDB family NZ13-MGT by GTDB-tk (Chaumeil et al., 2020) were assembled from metagenomes from terrestrial and marine hydrothermal systems globally.

Importance of multiple genomes per species. It is recommended (main text Table 2) that species or subspecies named under the SeqCode include more than one genome. This parallels the general recommendation under the ICNP to characterize multiple strains for proposals of new taxonomic names and is especially important for MAGs and SAGs because of challenges associated with accurately binning metagenomic data and the low completeness of most SAGs. Here, FastANI was used to dereplicate the 78 high-quality MAGs into 11 >95% ANI clusters (i.e., species clusters), and phylogenetic analyses of concatenated marker gene sets confirmed that each ANI cluster was monophyletic. In total, nine of the species clusters were represented by multiple high-quality MAGs derived from metagenomes from different sampling dates and/or geothermal springs, ranging from two to 16 MAGs. Comparison of the multiple MAGs per species cluster allowed us to assess: (1) monophyly of the species by using a multiple marker gene set; (2) the true presence of multiple copies of normally single-copy, conserved marker genes and true absence of conserved marker genes used to assess genome completeness and contamination; (3) the existence of homologs of genes encoding important functions (in this case, tungstate transporters, tungstoenzymes, and genes related to energy conservation) to support the association of those genes/pathways with the species; (4) shared gene content in general; and (5) similar genome sizes for the genomes within a species. These comparisons strengthened our conclusions about the proposed mode of energy conservation and evolution of the organisms and allowed us to identify and reject problematic MAGs. Briefly, various bioinformatic pipelines were used for generation of MAGs, but metagenomic assembly was typically performed with metaSPAdes (Nurk et al., 2017), followed by binning of contigs > 2.5 kbp in length with the MetaBAT (Kang et al., 2015) or MetaBAT2 (Kang et al., 2019), and/or MaxBin2 (Wu et al., 2016), and/or CONCOCT v.1.1.0 (Alneberg et al., 2014) algorithms. In some cases, the "bin_refinement" module of MetaWRAP was also used to identify the highest quality MAGs among the three binning strategies, and quality

of all genomes was assessed based on CheckM v.1.1.39 estimates of completeness and contamination. Only those MAGs exhibiting > 90 % estimated completeness and < 5 % estimated contamination are reported here. Here, we rejected all MAGs generated by MaxBin2 (Wu et al., 2016) because all were massively contaminated, as evidenced by reproducibly larger and more variable genome sizes than MAGs generated with other pipelines, but without any additional conserved marker genes. These MAGs were therefore not flagged as contaminated by automated contamination detection software such as CheckM (Parks et al., 2018), so comparison of the MAGs within each species was critical.

In this study, we named two species based on single high-quality MAGs. In one case, *Terraquivivens tikiterensis*, we justified this decision based on the very high quality of the MAG (99.0 % completeness with 0.49 % estimated contamination; 75x coverage including sequences for 5S, 16S and 23S rRNAs and tRNAs coding for 19 amino acids) and the availability of 24 MAGs from other species of the genus for comparison. In the other case, *Benthortus lauensis*, we justified this decision again on the high quality of the MAG (94.0 % completeness with 2.91 % estimated contamination; 34x coverage including sequences or 5S, 16S and 23S rRNAs and tRNAs coding for 20 amino acids) and on the difficulty of obtaining additional samples from the only known habitat of this genus, the Lau Hydrothermal Field.

Data quality under the SeqCode. Only MAGs exhibiting estimated completeness > 90 % and estimated contamination < 5 % were considered possible candidates for naming under the SeqCode. We furthermore accounted for rRNAs and tRNAs and selected the best MAG representing each species cluster as type genome, ensuring that we adhered to data quality standards (Table 3 of the main text). We note that several GTDB species representatives did not contain any high-quality MAGs; those MAGs were analyzed phylogenetically in our study, but they could not be named under the SeqCode. Nevertheless, they can be identified using GTDB placeholder names.

Nomenclature under the SeqCode. We decided to propose names for all 11 species clusters under the SeqCode as well as the parent taxa as prescribed under the SeqCode. Names were formed under the rules of Latin following general recommendations of Appendix 9 in the ICNP and were checked by the nomenclature expert Aharon Oren. To follow Rules 18a and 26 in the SeqCode, the publication includes the following for each taxonomic name that is proposed: (i) clear designation of their nomenclatural type; (ii) designation of the taxonomic rank; and (iii) the etymology of the new name (Buessecker et al., 2022). We also followed Recommendation 26 in the SeqCode by clearly separating the nomenclature proposals in the manuscript within a protologue. An annotated example protologue (with annotations [*in brackets in italics*]) for each taxonomic rank is shown below, preceded by a brief discussion of the elements of the protologue. To be clear, standardized protologues are not required under the SeqCode as long as the rules of the SeqCode are applied, particularly Rule 18a and Rule 26 for new taxon names that don't require unions or divisions of existing taxa. Authors of publications proposing many new taxa may choose to summarize the required elements as a table, for example. A summary of the key elements required by the SeqCode are listed in Supplementary Table S1 for these same three taxonomic names as an example.

Example protologue for a new family. The protologue below describes the new family *Wolframiiraptoraceae*. Note that for a family or higher taxon, the nomenclatural type is a genus. Here, that genus is *Wolframiiraptor* from which the name of the family must be derived under Rule 15 of the SeqCode.

Description of the family *Wolframiiraptoraceae*

Wolframiiraptoraceae (Wolf.ra.mi.i.rap.to.ra'ce.ae. N.L. masc. n. *Wolframiiraptor*, type genus of the family; L. fem. pl. suff. *-aceae*, ending to denote a family; N.L. fem. pl. n. *Wolframiiraptoraceae*, family of the genus *Wolframiiraptor*). *[This text designates the taxonomic rank (family) and the etymology under Rule 26.4 and 26.5. Both will also be captured in defined fields in the SeqCode Registry.]*

Members of this family are associated with thermal aquatic environments and have been identified from geothermal springs in China, New Zealand and the USA and a marine hydrothermal vent in the Western Pacific. Phylogenomic inference robustly recovers the genomes of these organisms as a well-supported monophyletic lineage within the order *Caldarchaeales*, and delineation of these taxa as a family is supported by Relative Evolutionary Divergence (RED) and Average Amino Acid Identity (AAI). AAI values among designated type genomes for species in this family range between 65 and 85 % within proposed genera, and between 49 and 57 % among members of different genera. The distribution of genes required for oxidative phosphorylation indicate that members of the family may either be strict or facultative anaerobes. Sulfide-dependent respiration may also occur in some members of the family, but this trait is not conserved for all genera. Several putative tungsten-dependent ferredoxin oxidoreductases, specifically aldehyde ferredoxin oxidoreductases (AORs), formaldehyde ferredoxin oxidoreductases (FORs), and glyceraldehyde-3-phosphate ferredoxin oxidoreductases (GAPORs) are encoded by genomes belonging to this family. *[This text includes a description of the taxon, following Recommendation 26. Such text is recommended but not required under the SeqCode.]*

The nomenclatural type for the family is the genus *Wolframiiraptor*. *[This text designates the nomenclatural type under Rule 26.3. Note that the nomenclatural type for ranks of class, order, and family is the first legitimately named genus in the taxon. These dates are clearly shown in the SeqCode Registry. Rule 26.3 embodies Principle 5 and serves to unambiguously identify the taxon. See Rule 16, especially Table 2, Rule 18, and Rule 22. Under the SeqCode, the name of newly proposed higher taxon must be derived from the root of the type genus* Wolframiiraptor *(*Wolframi(-um*)) plus a connecting vowel (i),* raptor *(genitive case* –oris*, dropping the ending of genitive case gives us the root* raptor-*) followed by the appropriate suffix to denote the rank (-*aceae*). See Rule 15 and Table 1.]*

Example protologue for a new genus. The protologue below describes the new genus *Wolframiiraptor*. Note that for a genus, the nomenclatural type is a species.

Description of the genus *Wolframiiraptor*

Wolframiiraptor (Wolf.ra.mi.i.rap'tor N.L. neut. n. *wolframium*, tungsten; L. masc. n. *raptor*, snatcher or thief; N.L. masc. n. *Wolframiiraptor*, snatcher of tungsten). *[This text designates the taxonomic rank (genus) and the etymology under Rule 26.4 and 26.5.]*

Members of this genus have been identified from geothermal springs from the Great Basin, Yellowstone National Park, USA, and the Rehai Geothermal Field and the town of Dientan, Tengchong, China. AAI values among genomes representing separate species within the genus range between 81 and 90 %. Based on ancestral state reconstruction analysis, likely losses of the genes encoding cytochrome c oxidase subunits, the aerobic carbon-monoxide dehydrogenase large subunit, and sulfide:quinone oxidoreductase (Sqr), indicate that members of this genus are likely strict anaerobes and are incapable of sulfide oxidation. Genomes of this genus encode a *tupA* subunit of the tungstate (Tup) ABC transporter and contain multiple genes encoding tungsten-dependent oxidoreductases, including three putative AOR-like, one FOR-like and one GAPOR-like proteins. This taxon is supported as a genus-level group by phylogenomics, AAI and RED. *[This text includes a description of the taxon, following Recommendation 26. Such text is recommended but not required under the SeqCode.]*

The nomenclatural type of the genus is *Wolframiraptor gerlachensis*^{Ts}. *[This text designates the nomenclatural type under Rule 26.3. Note that the nomenclatural type for rank of genus is a species, typically the first legitimate species in the genus. These dates are clearly shown in the SeqCode Registry. Rule 26.3 embodies Principle 5 and serves to unambiguously identify the taxon. See Rule 16, especially Table 2, and Rule 22. Note that genus names do not need to have a standard suffix like family, order and above, but they should avoid suffixes used for other taxonomic ranks to prevent confusion. See Rule 15 and Table 1. Under Chapter 4, the superscript Ts can be added when this species is a nomenclatural type under the SeqCode.]*

Example protologue for a new species. The protologue below describes the new species *Wolframiiraptor gerlachensis*. Note that for a species, the nomenclatural type is a DNA sequence, typically a genome assembly (Table 2).

Description of *Wolframiiraptor gerlachensis*^{Ts}

Wolframiiraptor gerlachensis (ger.lach.en'sis N.L. masc. adj. *gerlachensis*, of Gerlach, the region where Great Boiling Spring is located in Nevada, and where the samples containing this organism were obtained). *[This text designates the taxonomic rank (species) and the etymology under Rule 26.4 and 26.5. Under Chapter 4, the superscript Ts can be added to denote that this species is the type for the genus.]*

A MAG representing this species was recovered from metagenomic sequencing of a stable enrichment culture, established from an *in-situ* corn stover enrichment from Great Boiling Spring, Nevada, USA. Enrichment and maintenance of this species within the mixed-culture community

was optimal at an incubation temperature of 80 °C with lignocellulose and sugars, at circumneutral pH. This species is dependent on tungsten for growth, with significant decline in its abundance within the community without tungsten added to the growth medium. Additionally, several tungstoenzymes conserved within the genus were expressed at a higher rate during growth on corn stover, suggesting direct involvement of tungstoenzymes in complex carbohydrate metabolism. Cells of this organism showed significant isotope enrichment when grown on isotopically labeled xylose-amended medium, with limited isotope enrichment during growth on medium amended with amino acids, glucose, ribose, and starch, indicating preferential assimilation of xylose. The genome sequence for the type of this species is 1,277,965 bp, consists of 27 contigs, and has a G+C content of 52 %. Completeness is estimated at 98.06 % with 0.49 % contamination, as estimated with CheckM. ANI comparisons among this genome and those of closely related species were below 86 %, supporting the delineation of this taxon as unique and distinct to other species in the genus. *[This text includes a description of the taxon, following Recommendation 26. Such text is recommended but not required under the SeqCode.]*

The genome Wger_A8^{Ts}, available under the GenBank assembly accession number (GCA_021323375.2^{Ts}), is the designated nomenclatural type for the species, and was recovered from an enrichment culture, established from an *in-situ* enrichment from Great Boiling Spring, Nevada, USA. *[This text designates the nomenclatural type under Rule 26.3. Note that the nomenclatural type for rank of species or subspecies is a DNA sequence, typically a genomic assembly (Table 1). Rule 26.3 embodies Principle 5 and serves to unambiguously identify the taxon. Metadata for this sequence is included in the GenBank entry. Under Chapter 4, the superscript Ts can be added to denote that this genomic assembly is the nomenclatural type of the species.]*

Supplementary Table S1. Example spreadsheet format for nomenclature proposals. Example proposals for family, genus, and species ranks. Such tables are simple alternatives to protologues and may be especially valuable when many names are proposed.

**Proposed taxon**	**Etymology**	**Nomenclatural type**
Family *Wolframiiraptoraceae*	Wolf.ra.mi.i.rap.to.ra'ce.ae N.L. masc. n. *Wolframiiraptor*, type genus of the family; L. fem. pl. suff. *-aceae*, ending to denote a family; N.L. fem. pl. n. *Wolframiiraptoraceae*, family of the genus *Wolframiiraptor*	Genus *Wolframiiraptor*
Genus *Wolframiiraptor*	Wolf.ra.mi.i.rap'tor N.L. neut. n. *wolframium*, tungsten; L. masc. n. *raptor*, snatcher or thief; N.L. masc. n. *Wolframiiraptor*, snatcher of tungsten	Species *Wolframiiraptor gerlachensis*^{Ts}
Species *Wolframiiraptor gerlachensis*^{Ts}	ger.lach.en'sis N.L. masc. adj. *gerlachensis*, of Gerlach, the region where Great Boiling Spring is located in Nevada, and this organism was obtained from	Genomic assembly: GCA_021323375.2^{Ts}

SeqCode Pre-registration: registration of path 1 names in the expert mode of the SeqCode Registry

Names may currently be entered into the SeqCode Registry (https://seqco.de/) using the expert mode. Guided submission modes are also under development to assist users with the registration process. Within expert mode, a profile is created in the SeqCode Registry (https://seqco.de/) and then contributor privileges are requested. Once approved, a "Contributor" dashboard will become available. The "Create a name" option for name registration is selected. Then the proposed name is provided in the textbox and "Register" is selected (e.g., *Wolframiiraptor gerlachensis* as an example). This will only reserve the name and does not entail validation. During this registration step, automated quality checks will be performed, and red and orange flags will be generated with descriptions of what is required or recommended, respectively, and how each relates to Rules and Recommendations of the SeqCode. Red flags need to be addressed before progressing further. For "Missing rank" red flags, "Define rank" is selected, and then the appropriate rank is selected (e.g., species), and "Submit" is selected. For addressing the "Missing type" red flags, select "Edit type", and under material, select the appropriate description of the type genome sequence and provide its accession number (e.g., GenBank assembly; GCA_021323375.2). Here additional orange flags relating to the genome serving as nomenclatural type can also be addressed. For the "Missing genome kind" the type of genome can be selected (e.g., Metagenome-assembled genome (MAG))

and the sequencing depth can be indicated (e.g., 12 in the case of GCA_021323375.2). If available, provide the source information under the "Missing genome source" orange flag (e.g., INSDC Sequence Read Archive (SRA) with the run accessions SRX13128468 and SRX13128467, comma-separated for multiple run accessions). Additionally, to assist in genome quality assessments of genomes by curators, indicate the quality metrics in this section (e.g., G+C content (%), estimated completeness and contamination, the completeness fraction of the 16S and 23S rRNA genes, and how many rRNA fragments and predicted tRNA elements are present in the genome). To add the etymology, select "Edit etymology" under the "Missing etymology" red flag. Provide the syllabication and grammar information of components of the name (e.g., ger.lach.en'sis N.L. masc. adj. *gerlachensis*, of Gerlach, the town where Great Boiling Spring is located in Nevada, and where the samples containing this organism were obtained). To address additional orange flags, the same process can be followed, although these flags are not obligatory for registration. Orange flags can include, but are not limited to, "Missing description", "Missing parent", and "Missing effective publication" flags. If a description for the taxon is available, add this to the "Missing description" flag (e.g., the description from the protologue included for publication). If possible, link a parent taxon to the name, even if the parent taxon is also a newly proposed name to be registered (e.g., *Wolframiiraptor*). As the pre-registration of the names is occurring before effective publication, the "Missing effective publication" flag remains. If additional names or parent taxon names are proposed, complete this process for all proposed taxa. This can be accessed and/or evaluated under the "My names" list in the "Contributor" dashboard. Once all required information for review is provided for each entry, the "Propose name" option under "Contributor Actions" can be selected for each entry. This allows the addition of the name to a new register list or, as part of larger projects with a single intended effective publication, all proposed taxa can be added to the same register list for curator review. Select "Register name" to generate a draft register list. This draft register list is assigned an accession URL that should be included in the effective publication (e.g., seqco.de/r:slp2ijs4). Submit the draft register list for curator review. Once a curator provisionally accepts the names, this completes pre-registration. At this point, the proposed names are hidden from the public but can be accessed via the URL, allowing reviewers and editors to review the proposed names.

Finally, authors are required to complete the registration/validation process by entering the DOI and submitting the published article, at which point the names become valid and visible to the public. Minor edits/corrections can be made at any time following validation by registered contributors as long as they are validated by curators. While curators will currently ensure the data quality while guiding users through the naming process; contingent upon community support and funding, automated checks will be implemented for evaluation of genome sequence quality and names.

Example of path 2: *Macondimonas diazotrophica*[Ts]

Path 2 (main text Figure 1) should be used to register and validate names under the SeqCode after the effective publication is already published. This could be done by the authors or by other scientists, although we recommend working with authors to register their names whenever feasible. The names of the taxa must appear in the effective publication including online supplementary material, although minor orthographic variants are allowable.

Overview. Several authors of the SeqCode (Konstantinidis, Rodriguez-R, Rosselló-Móra) were co-authors on a publication on the metagenome-guided isolation of a crude-oil degrading bacterium, abundant in oil-contaminated sediments of coastal marine ecosystems but largely absent from pristine environments, recovered specifically from the oiled sands of Pensacola Beach after the oil spill in April of 2010 in the Gulf of Mexico. The initial metagenomic datasets from the oiled sands (Rodriguez-R *et al* 2015) were used to identify a high-quality MAG through targeted genome reconstruction on the basis of dominant *nifH* gene alleles. The reconstructed MAG was used to reconstruct the metabolic potential of this species, identify auxotrophies, and ultimately isolate this organism (Karthikeyan et al 2019). The resulting isolate genome has a 99.8% ANI with the MAG, but the fastidious nature of the microorganism prevents its deposition in culture collections. In the publication (Karthikeyan *et al* 2019), the microorganism is identified as a novel genus in the order *Chromatiales*, designated *Macondimonas*. The only species and nomenclatural type of the genus is *Macondimonas diazotrophica*. The publication designates strain KTK01 as type material of the species, which we emend here to specify instead the genome sequence.

Best practices for cooperativity for Path 2. We consider it best practice to communicate with key authors before registering and validating effectively published names under the SeqCode. In this case, three of the authors of the original publication including the corresponding author* (Konstantinidis*, Rodriguez-R, Rosselló-Móra) registered the names in the SeqCode Registry.

Deposition of type DNA sequences for species and subspecies into an INSDC database. The genome designated as the nomenclatural type for the proposed species is publicly available from an INSDC database under the GenBank Assembly accession number GCA_004684205.1, in accordance with Rule 18a. This rule states that type DNA sequences must be available from INSDC databases, a decision made to promote stable access to the data.

Protologues and descriptions (Karthikeyan et al., 2019)

Description of *Macondimonas* gen. nov

Macondimonas (Ma.con.di.mo’nas. L. fem. n. *monas*, a unit, a monad; N.L. fem. n. *Macondimonas*, a monad from Macondo Prospect, the site of DWH oil spill. Additionally, Macondo is a fictional town in A Hundred Years of Solitude by G. G. Márquez. In the book, the town of Macondo has a rapid population growth, a period of economic prosperity, and then a rapid

population fall, which is reminiscent of the ecologic pattern observed for this group upon crude-oil exposure)

Members of this genus exhibit a coccobacilli morphology and a heterotrophic aerobic metabolism. No phototrophic, nor chemoautotrophic growth, or their corresponding genes in the genome were observed. The type species is *Macondimonas diazotrophica*.

Description of *Macondimonas diazotrophica* sp. nov.

Macondimonas diazotrophica (di.a.zo.tro'phi.ca. Gr. pref. *di*, in two; N.L. neut. n. *azotum*, nitrogen; N.L. pref. *diazo-*, pertaining to dinitrogen; Gr. adj. *trophikos* -ê -on, feeding, tending; N.L. fem. adj. *diazotrophica*, one that feeds on dinitrogen, named after its ability to fix atmospheric nitrogen)

Cells grown on solidified mineral artificial seawater media using hexadecane as substrate show a coccobacillus morphology, of about 0.6 µm in length and 0.35 µm in width, and formed circular colonies. Members of the species are aerobes, growing at a pH range of 6.5–8.5 with a pH optimum of 7.5, and a salinity range of 250–500 mM of NaCl, with an optimum concentration of 330 mM. The temperature range for optimal growth is 22–30 °C, with no growth observed at 4 °C and above 34 °C. Cells can grow with hexadecane and pyruvate as a sole carbon sources and fix nitrogen. Genome size is ~2.8 Mbp with a G+C% content of 61.56. The designated type material is strain KTK01, and its genome sequence can be found under NCBI BioSample accession number SAMN11302943.

Emendation of the species *Macondimonas diazotrophica*^{Ts}

The properties of the species are as given by Karthikeyan et al 2019, and the designated nomenclatural type is the genome of strain KTK01, deposited in the NCBI databases with accession number GCF_004684205.1.

Registration of effectively published names in the SeqCode Registry. Registration of names in the SeqCode Registry can occur after effective publication. This example outlines the registration of a name after effective publication through the expert registration mode in the Registry. Register as "Contributor". Select the "Create a name" option from the "Contributor" dashboard and provide the effectively published name in the textbox and select register (e.g., *Macondimonas diazotrophica*). Similar as described before, automatic checks are performed, and red and orange flags related to Rules and Recommendations in the SeqCode are generated. In addition to addressing all flags described before, the orange "Missing effective publication" flag needs to be addressed. Select the "Register publication" option under this flag, provide the DOI to the effective publication (10.1038/s41396-019-0400-5) in the provided space, and select "Register". From here, the linked publication shows which names are linked to the specific publication. Select the taxon name being edited (e.g., *Macondimonas diaozotrophica*). This publication will appear in the "Publications" section of the entry (end of page). Several publications proposing, emending,

correcting, or citing the taxon in question can be linked to the name in this fashion. Under the "Actions" associated with the publication, select "Proposed this name" for publications proposing the name, "Corrigendum" for publication correcting names or "Emended this name" for published emendations. Repeat this process for any higher-level taxonomic names and link the associated effective publication with these names. Notify the SeqCode curators by submitting these names (typically with all associated names in a single Register List) as a draft register list for curator review (e.g., seqco.de/r:6ns_rdob) and click "Validate Published Names" to submit the manuscript PDF and (if relevant) supplementary material. If proposed names satisfy all criteria for validation under the SeqCode, proposals will be reviewed, and contributors will be notified of validation under the SeqCode or further recommendations on corrections to the submission.

Example of combination of path 1 and path 2 for validation*:* class *Kryptonia* and child taxa

In some cases, some names are published in an effective publication, but additional names are also warranted, such as a case where one or more new higher ranks for a proposed taxon are not published. This would require a combination of path 1 (registration of new names) and path 2 (registration of effectively published names).

Overview. As an example for a combination of Path 1 and Path 2, Eloe-Fadrosh et al. (2016) proposed the new genus and species *Candidatus* Kryptonium thompsoni based on 13 SAGs and a single MAG from geothermal springs in British Columbia Canada, Yunnan Province China, and Nevada USA. In addition, *Candidatus* Kryptonium thompsoni was visualized by FISH in cells collected from Dewar Creek, British Columbia. *Candidatus* Kryptonium thompsoni was proposed as one of four *Candidatus* genera, each with a single species, belonging to a new candidate phylum named Kryptonia. None of the ranks between genus and phylum were named. In Supplementary Table 4 of that paper, the proposed candidate taxa were named, and the etymology described. Ranks were described in the manuscript. However, no nomenclatural type was designated because there was no path to validate the names under the ICNP or any other authority at the time the paper was published. Many problems like this exist in the past literature because the community hadn't come together to develop standards or best practices, and this is part of the impetus for the SeqCode. The SeqCode is deliberately designed to allow critical information to be captured in the SeqCode Registry, allowing most previous *Candidatus* names to be named under the SeqCode, provided the names appear in an effective publication. However, substantial changes to published names will need to be described separately in a peer-reviewed paper, which then becomes the effective publication. This test case is a good example, as described below.

Taxonomic decisions and example of taxonomic freedom. Per Principle 1, the SeqCode does not endorse any particular taxonomy. This is true of all major codes of nomenclature except the *International Code of Virus Classification and Nomenclature*. The taxonomic decisions described in this section were made by one of the authors of the original publication describing these genomes (Hedlund) and Palmer in collaboration with the first author (Eloe-Fadrosh) and

corresponding author (Ivanova). Again, we consider it best practice to communicate with key authors before registering and validating names under the SeqCode. It is our position that the four genera proposed in the effective publication should be combined into one genus based on (i) monophyly with strong support in phylogenetic analyses using conserved marker genes; (ii) RED values consistent with a single genus as implemented within GTDB; and (iii) average amino acid identity (AAI) values consistent with a single genus (>65% (Konstaninidis et al., 2017)). In this case, we will retain the genus and species name *Kryptonium thompsonii* for the largest group of genomes described from Dewar Creek. This is the largest species cluster, and it was also visualized by FISH. This MAG is of high quality per Genome Standards Consortium recommendations (Bowers et al., 2018) (18 contigs, 95.61 % completeness with 1.91 % estimated contamination; 68x coverage and sequences of the 5S, 16S and 23S rRNAs and tRNAs coding for 20 amino acids). Below is the original etymology from Supplemental Table 4 from the effective publication, but with the *Candidatus* prefix removed (Supplementary Table S2). This etymology with or without the *Candidatus* prefix and information contained in the paper is sufficient to satisfy Rules 26.1, 26.4, and 26.5 to name both the genus and species. Rules 26.2 and 26.3 will be satisfied when the name is registered with the SeqCode Registry. In this case, the nomenclatural type for the species will be the highest quality genome in the species cluster, which is the MAG JGI-4. The superscript ^{Ts} is added after the species epithet to denote that this is the type species of the genus, following Chapter 4. We also note that the species name has been corrected to *thompsonii*, as the second "i" is the correct suffix to denote a genitive masculine noun, in this case for a species named after a male (David Thompson). "Perfect" Latin is recommended under the SeqCode but not required. We consider this to be a minor orthographic variant on the original name. Also, the etymology is modified from the original to remove the definition of 'bios' because the root 'bios' does not appear in the name. We note here that the GTDB species representative (JGI-8) is actually a medium-quality MAG. We speculate that the GTDB used this species representative, a SAG, as it was erroneously labeled an "isolate" in NCBI, which was apparently carried over to GTDB. Thus, we recommend caution and care at all levels when designating nomenclatural types to ensure that the highest quality genomic data are used.

Modified etymology and typification of *Kryptonium thompsonii*^{Ts}. Eloe-Fadrosh et al., 2016 remains the effective publication.

Description of the genus *Kryptonium*

Kryptonium (Kryp.to'ni.um. Gr. adj. *krypton*, hidden; N.L. neut. n. *Kryptonium* a hidden life form).

The nomenclature type of the genus is *Kryptonium thompsonii*^{Ts}.

Description of the species *Kryptonium thompsonii*

Kryptonium thompsonii (thomp.son'i.i. N.L. gen. masc. n. after David Thompson, explorer of the region around Dewar Creek).

The nomenclatural type is GCA_001442925.1^{Ts}.

Following Recommendation 7, the ranks above the genus *Kryptonium* should be named until an existing name of a parent taxon exists. All available phylogenetic analyses of this genus indicate that it is very distant from the closest relatives named under the ICNP. However, there is disagreement between the original publication describing the SAGs and MAGs and the GDTB taxonomy as to whether the monophyletic lineage containing *Kryptonium* should be considered a phylum or a class. As a matter of conservatism, we choose to only propose names up to the rank of class as suggested by the GTDB taxonomy and ascribe that class to the phylum *Bacteroidota*. The GTDB has suggested the following names at ranks of family, order, and class: *Kryptoniaceae*, *Kryptoniales*, and *Kryptoniia*. Those names follow the rules of the SeqCode, particularly Rule 15, which states that the "The name of a family, order, class, or phylum is formed by the addition of the appropriate suffix to the stem of the type genus name". Thus, we will use the GTDB names but require an etymology, which is provided in the table below. The current manuscript then becomes the effective publication for those names (Path 1).

Table S2. Etymology and typification of higher taxa for *Kryptonium thompsonii*^{Ts}

**Proposed taxon**	**Etymology**	**Nomenclatural type**
Family *Kryptoniaceae*	Kryp.to.ni.a'ce.ae N.L. neut. n. *Kryptonium*, type genus of the family; L. fem. pl. suff. *-aceae*, ending to denote a family; N.L. fem. pl. n. *Kryptoniaceae*, family of the genus *Kryptonium*	Genus *Kryptonium*
Order *Kryptoniales*	Kryp.to.ni.a'les N.L. neut. n. *Kryptonium,* type genus of the order; N.L. fem. pl. suff. *-ales*, ending denoting an order; N.L. fem. pl. n. *Kryptoniales*, order of the genus *Kryptonium*	Genus *Kryptonium*
Class *Kryptoniia*	Kryp.to'ni.i.a N.L. neut. n. *Kryptonium*, type genus of the class; N.L. neut. n. suff. *-ia*, ending to denote a class; N.L. neut. pl. n. *Kryptoniia*, class of the genus *Kryptonium*	Genus *Kryptonium*

Additional species of the genus *Kryptonium*. As described above, we have chosen to merge four genera originally proposed into the single genus *Kryptonium*. In the GTDB, these genomes are instead divided into six species clusters based on circumscription radii determined from ANI

clustering, splitting the original taxon *Candidatus* Kryptobacter tengchongensis into three species. However, these two additional species clusters likely represent artificial clusters with very high sequence similarity because these clusters are based on three replicate MAG assemblies each. Regardless, only one other species cluster has a high-quality genome representative that could serve as a type under minimal standards described in Table 2 (main text). Thus, we will only name a single additional species for the cluster originally proposed as *Candidatus* Kryptothermus mobilis, from Great Boiling Spring, Nevada, USA. This taxon was enriched *in situ* in Great Boiling Spring sediments following the addition of corn stover, consistent with many annotated glycoside hydrolases in the genome. The original publication has only one MAG; the GTDB cluster again has three MAGs, although all are publicly available replicate assemblies from the same metagenomic sequencing run. We nevertheless choose to name this species based on the high quality of the MAG (97.25 % completeness with 2.73 % estimated contamination; 44x coverage and sequences of 5S, 16S and 23S rRNAs and tRNAs coding for 20 amino acids) and based on recruitment of the genome at high identity in two different cellulolytic enrichments in Great Boiling Spring that were incubated at 77 °C and 85 °C, providing clear evidence of growth at in situ temperature and consistent with a role in consortial degradation of plant biomass. In addition, 21 MAGs and SAGs from different samples representing the genus are available for comparison, providing confidence of genome size, assembly fidelity, and the presence/conservation of key genes.

In the case of the union of the genus *Kryoptonium* and *Candidatus* Kryptothermus, we retain the genus name *Kryoptonium*, as justified above. However, we consider this a nom. nov. because not only is a different genus name used, but also the species name should be changed.

Etymology and typification of *Kryptonium mobile* nom. nov.

Kryptonium mobile **(**mo'bi.le. L. neut. adj. *mobile*, motile, moving).

Synonym: *Candidatus* Kryptothermus mobilis (Eloe-Fadrosh et al., 2016)

The nomenclatural type is NCBI Assembly GCA_900070205.1.

Pre-registration of names in the SeqCode Registry. To register names for taxa that encompass names for a lineage where some were proposed in the effective publication and others that are new (and have been effectively published), register as a "Contributor" to the SeqCode Registry. Select "Create a name" option on the "Contributor" dashboard and register all names associated with the lineage as described above in the *Wolframiiraptoraceae* example (e.g., *Kryptoniia*, *Kryptoniales*, *Kryptoniaceae*, *Kryptonium*, *Kryptonium thompsonii*, and *Kryptonium mobile*). Link effective publications for the taxon names proposed previously (e.g., *Kryptonium thompsonii* proposed by Eloe-Fadrosh et al., (2016) [10.1038/ncomms10476], corrected by Oren et al., (2020) [10.1099/ijsem.0.003789]) and create new names for those that have not been proposed before (e.g., *Kryptoniia*, *Kryptoniales*, *Kryptoniaceae*, and *Kryptonium mobile*). As these taxa would have different effective publications associated with the taxon names, separate register lists need to be

created and associated with the appropriate effective publications. Add all of the taxa associated with the previously published effective publication to a single register list for approval by a curator (e.g., seqco.de/r:jvapsuy2) and all newly proposed names to a separate register list (e.g., seqco.de/r:rgrmqbgb). The provided register list accession URL for the newly proposed names should be included in the effective publication to allow automated checks and validation upon publication if approval was given by the curator. In this case, this manuscript serves as the effective publication for *Kryptoniia*, *Kryptoniales*, *Kryptoniaceae*, and *Kryptonium mobile*, and should be provided for validation under "Validate Published Names" in PDF along with supplementary material (if relevant).

References

1. Alneberg, J., et al. Binning metagenomic contigs by coverage and composition. *Nature Methods* **11**, 1144–1146 (2014).
2. Beussecker S, Palmer M, Lai D, Dimapilis J, Mayali X, Mosier D, Jiao J-Y, Colman DR, Keller LM, St. John E, Miranda M, Gonzalez C, Gonzalez L, Sam C, Villa C, Zou M, Bodman N, Robles F, Boyd ES, Cox AD, St. Clair B, Hua Z-S, Li W-J, Reysenbach A-L, Stott MB, Weber PK, Pett-Ridge J, Dekas AE, Hedlund BP, Dodsworth JA. Tungsten is essential for carbohydrate metabolism in members of a novel family of anaerobic, thermophilic *Caldarchaeales* (Aigarchaeota). *Nature Communications*, in press (2022).
3. Chaumeil, P.-A., Mussig, A. J., Hugenholtz, P. & Parks, D. H. GTDB-Tk: a toolkit to classify genomes with the Genome Taxonomy Database. *Bioinformatics* **36,** 1925-1927 (2020).
4. Eloe-Fadrosh EA, Paez-Espino D, Jarett J, Dunfield PF, Hedlund BP, Dekas AE, Grasby SE, Brady AL, Dong H, Briggs BR, Li WJ, Goudeau D, Malmstrom R, Pati A, Pett-Ridge J, Rubin EM, Woyke T, Kyrpides NC, Ivanova NN. Global metagenomic survey reveals a new bacterial candidate phylum in geothermal springs. *Nature Communications*. 7:10476. (2016). https://doi.org/10.1038/ncomms10476
5. Hua ZS, Wang YL, Evans PN, Qu YN, Goh KM, Rao YZ, Qi YL, Li YX, Huang MJ, Jiao JY, Chen YT, Mao YP, Shu WS, Hozzein W, Hedlund BP, Tyson GW, Zhang T, Li WJ. Insights into the ecological roles and evolution of methyl-coenzyme M reductase-containing hot spring archaea. *Nature Communications* 10:4574. (2019).
6. Kang, D. D, Froula, J., Egan, R., & Wang, Z. MetaBAT, an efficient tool for accurately reconstructing single genomes from complex microbial communities. *PeerJ* **3**, e1165 (2015).
7. Kang, D. D., et al. MetaBAT 2: an adaptive binning algorithm for robust and efficient genome reconstruction from metagenome assemblies. *PeerJ* **7**,e7359 (2019).
8. Karthikeyan, S., Rodriguez-R, L.M., Heritier-Robbins, P. *et al.* "*Candidatus* Macondimonas diazotrophica", a novel gammaproteobacterial genus dominating crude-oil-contaminated coastal sediments. *ISME J* **13,** 2129–2134 (2019). https://doi.org/10.1038/s41396-019-0400-5

9. Li, D., Liu, C. M., Luo, R., Sadakane, K., & Lam, T. W. MEGAHIT: an ultra-fast single-node solution for large and complex metagenomics assembly via succinct de Bruijn graph. *Bioinformatics* **31**, 1674-1676 (2015).
10. Nurk, S., Meleshko, D., Korobeynikov, A., & Pevzner, P. A. metaSPAdes: a new versatile metagenomic assembler. *Genome Research* **27**, 824-834 (2017).
11. Parks, D. H., Imelfort, M., Skennerton, C. T., Hugenholtz, P. & Tyson, G. W. CheckM: assessing the quality of microbial genomes recovered from isolates, single cells, and metagenomes. *Genome Research* **25,** 1043–1055 (2015).
12. Rodriguez-R, L., Overholt, W., Hagan, C. *et al.* Microbial community successional patterns in beach sands impacted by the Deepwater Horizon oil spill. *ISME J* **9,** 1928–1940 (2015). https://doi.org/10.1038/ismej.2015.5
13. Wu, Y.-W., Simmons, B. A., & Singer, S. W. MaxBin 2.0: an automated binning algorithm to recover genomes from multiple metagenomic datasets, *Bioinformatics* **32**, 605–607 (2016).
14. Uritskiy, G. V., DiRuggiero, J., & Taylor, J. MetaWRAP-a flexible pipeline for genome-resolved metagenomic data analysis. *Microbiome* **6**, 158 (2018).
